# Upadacitinib Restrains the Pathogenic Fitness of CD4^+^ T Cells and Aberrant B Cell Programming in Optic Neuritis

**DOI:** 10.1002/advs.77701

**Published:** 2026-09-09

**Authors:** Gengchen Jiang, Qi Jiang, Lian Han, Yue Peng, Xingyu Liu, Tong Yang, Hao Huang, Jiaying Liu, Yuan Gao, Hui Yang, Wenjun Zou, Zhaohuai Li, Wenru Su

**Affiliations:** ^1^ Department of Ophthalmology Ninth People's Hospital Shanghai Jiao Tong University School of Medicine Shanghai China; ^2^ State Key Laboratory of Eye Health Department of Ophthalmology Shanghai Ninth People's Hospital Shanghai Jiao Tong University School of Medicine Shanghai China; ^3^ The First Affiliated Hospital of Bengbu Medical University Bengbu Medical University Bengbu China; ^4^ School of Clinical Medicine Qinghai University Xining China; ^5^ Department of Ophthalmology Zhuzhou Hospital Affiliated to Xiangya School of Medicine Central South University Zhuzhou China; ^6^ State Key Laboratory of Ophthalmology Zhongshan Ophthalmic Center Guangdong Provincial Key Laboratory of Ophthalmology and Visual Science Sun Yat‐sen University Guangdong Provincial Clinical Research Center for Ocular Diseases Guangzhou China; ^7^ Department of Ophthalmology Wuxi No.2 People's Hospital Jiangnan University Medical Center Wuxi China

**Keywords:** autoimmune diseases, MCL1, optic neuritis, t helper 17 cells, upadacitinib

## Abstract

Optic neuritis (ON) is a neuroinflammatory autoimmune disease harboring autoreactive lymphocytes. Effector memory CD4^+^ T cells (CD4^+^ *T*_em_) represent a prominently expanded and pro‐inflammatory subset within this compartment, yet their pathogenic metabolic programs remain poorly defined. B cell dysfunction also contributes to ON pathogenesis. However, whether T cell‐intrinsic metabolic rewiring directly fuels this B cell dysregulation awaits elucidation. Here, through single‐cell transcriptomic profiling of peripheral blood mononuclear cells (PBMCs) from ON patients, we conceptualized a pathological circuit linking T cell metabolic reprogramming to aberrant T‐B crosstalk. In pathogenic CD4^+^ *T*_em_, JAK1 upregulation and enhanced STAT3 phosphorylation propagated a metabolic rewiring transcriptional program and secured T cell fitness via MCL1 induction. An inferred cholesterol export signature distinguished this subset and engaged the nuclear sensor RORA on B cells to instruct pro‐inflammatory polarization and humoral commitment. In turn, subverted B cells perpetuated exaggerated antigen presentation and cytokine secretion, cementing pathogenic CD4^+^ *T*_em_ differentiation and sustaining a self‐amplifying inflammatory loop. Cross‐disease profiling extended this axis to allied autoimmune disorders. Selective JAK1 blockade with upadacitinib (UPA) restored immune homeostasis and attenuated experimental autoimmune encephalomyelitis (EAE), phenocopied by MCL1 inhibition. Together, these findings nominate UPA as a targeted therapy for ON and allied autoimmune disorders.

## Graphical abstract

Single‐cell profiling and functional perturbation reveal coordinated JAK1‐pSTAT3 downstream programs in optic neuritis, including MCL1‐dependent fitness of pathogenic CD4^+^ *T*_em_ cells and glycolysis‐linked, cholesterol‐sensitive B‐cell responses associated with RORA. Upadacitinib disrupts this reciprocal T‐B‐cell circuit and alleviates neuroinflammation, thereby linking adaptive immune remodeling to a targetable metabolic axis and suggesting a new therapeutic direction for optic neuritis.

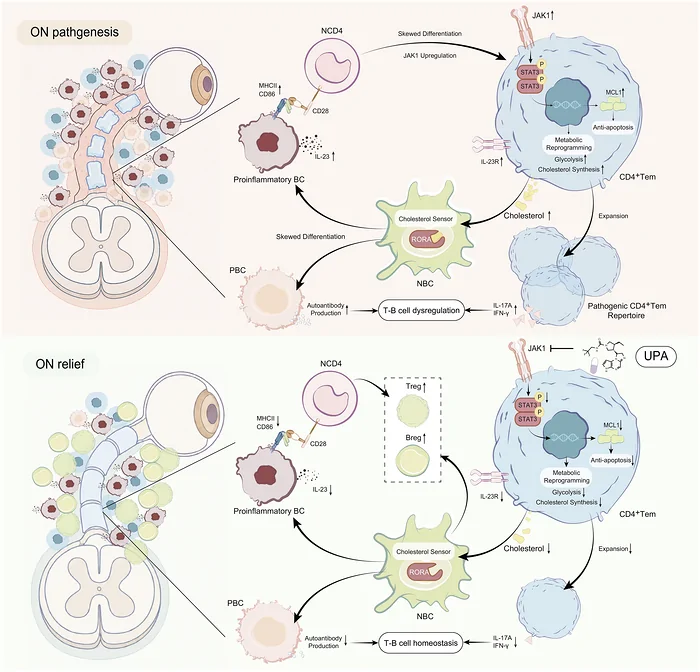

## 1 Introduction

Optic neuritis (ON) is an acute inflammatory demyelinating condition of the optic nerve and a frequent inaugural manifestation of central nervous system (CNS) autoimmune demyelinating disorders, including multiple sclerosis (MS), neuromyelitis optica spectrum disorder (NMOSD), and myelin oligodendrocyte glycoprotein antibody‐associated disease (MOGAD) [1]. Among these, MS represents the most prevalent etiology of ON [2, 3]. Nearly half of MS patients experience ON, which often antedates classical motor deficits and reflects the heightened susceptibility of the richly myelinated visual pathway to inflammatory injury, culminating in abrupt and often severe visual loss [4]. While partial recovery occurs in many patients, a considerable subset endures persistent visual disability due to irreversible neuroaxonal injury. Current therapy hinges on high‐dose glucocorticoids to hasten visual recovery, yet this approach offers no definitive long‐term visual benefit nor reduces the likelihood of progression to clinically definite MS [5, 6]. The persistence of corticosteroid‐refractory cases and recurrent episodes further underscores a critical therapeutic void [7]. Thus, novel targeted therapies represent an urgent priority.

Autoreactive CD4^+^ T cells are widely recognized as critical mediators that initiate and perpetuate the pathology of ON and its canonical animal model, experimental autoimmune encephalomyelitis (EAE) [8, 9]. Upon activation in peripheral lymphoid organs, these cells traffic across the blood–brain barrier and orchestrate a cascade of inflammatory events within the CNS. In particular, effector memory CD4^+^ T (CD4^+^ *T*_em_) cells are prominently expanded in ON patients and exhibit a pronounced pro‐inflammatory skew [10]. It is increasingly appreciated that the acquisition of such pathogenic effector functions is intimately coupled to profound metabolic reprogramming [11]. Whereas quiescent naïve T cells rely predominantly on oxidative phosphorylation (OXPHOS) and fatty acid oxidation (FAO) to meet their limited bioenergetic demands, activated effector populations undergo a glycolytic shift that supports rapid proliferation and cytokine production [12]. Whether and how this metabolic rewiring is achieved in pathogenic CD4^+^ *T*_em_ cells during ON, and whether it extends beyond cell‐intrinsic adaptation to influence the behavior of other immune lineages, remain poorly defined. In addition, B cells undergo significant alterations in ON, characterized by a loss of regulatory subsets and the expansion of pro‐inflammatory and autoantibody‐producing populations [13, 14]. In turn, this pro‐inflammatory shift reciprocally amplifies Th1 and Th17 responses [15]. The signals that drive this pathogenic B cell activation and antibody secretion, and the extent to which they are coupled to cues derived from autoreactive T cells, are incompletely understood. Therefore, a comprehensive dissection of the T‐B cell interplay and its metabolic underpinnings in ON would be of considerable value.

Upadacitinib (UPA) is an oral, reversible JAK1‐selective inhibitor that has demonstrated robust clinical efficacy and an acceptable safety profile across a growing spectrum of autoimmune diseases. Initially approved for rheumatoid arthritis, its therapeutic indications have since expanded to include psoriatic arthritis, atopic dermatitis, ankylosing spondylitis, ulcerative colitis, and most recently giant cell arteritis (GCA) [16, 17, 18, 19]. This capacity stems from the fact that JAK1 functions as a preferential signaling kinase downstream of multiple pro‐inflammatory cytokine receptors, including those for IL‐6 and type I interferons, and is critically required for the differentiation and maintenance of pathogenic effector T cell subsets [20]. Heightened JAK‐STAT pathway activity has been documented in a spectrum of CNS autoimmune disorders, suggesting that this signaling node may contribute to the pathogenic differentiation of autoreactive CD4^+^ T cells [21]. However, whether pharmacological JAK1 inhibition with UPA confers therapeutic benefit in neuroinflammatory disorders, particularly ON, has not been established, and the mechanisms by which JAK1 coordinates pathogenic T cell responses in this context remain enigmatic.

MCL1, a member of the BCL‐2 family of anti‐apoptotic proteins, has long been recognized as a critical gatekeeper of cell survival through its ability to sequester pro‐apoptotic effectors such as BAK and BAX [22]. Notably, its role in the immune system is emerging, as MCL1 is essential for activated T cell survival and the prevention of metabolic stress in CD4^+^ T cells [23, 24]. Whether MCL1 contributes to the persistence of pathogenic T cell populations in neuroinflammatory conditions, and whether its activity is selectively engaged in specific autoimmune contexts, has not been clarified.

In this study, we first employed single‐cell transcriptomic profiling of PBMCs from patients with ON to holistically depict the dysregulated immune landscape and uncover key pathogenic programs. These analyses revealed a pronounced bias in CD4^+^ *T*_em_ fate acquisition, underpinned by a coordinated shift in metabolic identity and enhanced apoptotic resistance. Moreover, a vicious loop instructed by aberrant T‐B cell crosstalk emerged, wherein cholesterol exported by CD4^+^ *T*_em_ cells engaged RORA on B cell compartments to propagate inflammatory signaling, and reciprocally, subverted B cells reinforced the pathogenic differentiation of CD4^+^ *T*_em_ cells. Central to this pathogenic network, we established the JAK1‐pSTAT3‐MCL1 axis as a pivotal regulator of this aberrant remodeling. In addition, cross‐disease single‐cell profiling extended the relevance of this axis to allied autoimmune settings, including GCA and Kawasaki disease (KD). Pharmacological blockade of JAK1 with UPA dismantled this circuitry and attenuated disease severity in the EAE model. Collectively, these results defined the JAK1‐pSTAT3‐MCL1 axis as a conserved pathogenic driver across ON and related autoimmune conditions, and validated UPA as a promising targeted therapeutic strategy for neuroinflammatory disorders.

## 2 Results

### 2.1 Single‐Cell Landscape Captures Immune Cell Dynamics in ON

**FIGURE 1:**
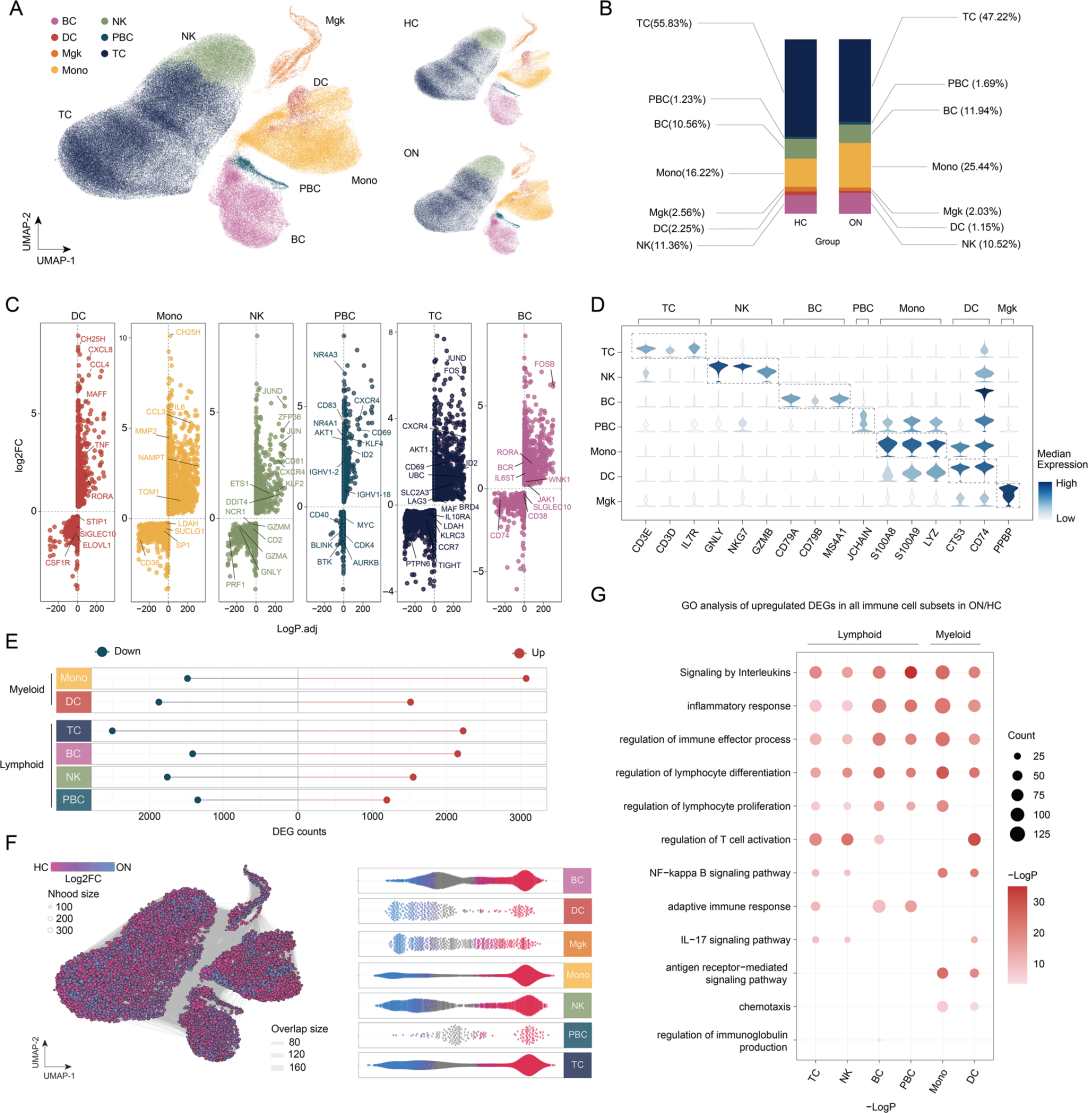
Single‐cell atlas of PBMCs from ON patients reveals pronounced TC activation and immune heterogeneity: (A) UMAP projections of all cells (left) and separated by HC and ON groups (right), colored by major immune lineages. (B) Bar plots showing the proportion of each major cell subset in HC and ON groups. (C) Volcano plots of ON‐DEGs for each major cell type. (D) Violin plots illustrating how each major immune cell subset is defined. (E) Lollipop charts of upregulated and downregulated DEG counts per major immune cell group. (F) MiloR analysis of ON versus HC samples across all immune cells. (G) GO enrichment analysis of upregulated ON‐DEGs across all cells.

To delineate the immune dynamics associated with ON, we performed an integrative reanalysis of single‐cell RNA‐seq data derived from PBMCs of ON patients and matched healthy controls (HC). Based on previously reported cell markers, all profiled cells were classified into 7 clusters: B cell (BC), plasma cell (PBC), T cell (TC), natural killer cell (NK), monocyte (Mono), dendritic cell (DC), and megakaryocyte (Mgk) (Figure 1A,D; Figure S1A,B and Table S1). To assess intra‐cluster heterogeneity, we also applied the ROGUE algorithm [25]. The desirable ROGUE scores obtained for all major cell types indicate low internal heterogeneity within each predefined population, thereby validating the robustness of our initial classification and affirming the distinctness and homogeneity of these populations for subsequent analysis (Figure S1D). We then observed a distinct compositional shift in the immune cell repertoire of ON, marked by the expansion of the Mono and BC compartments and the concomitant contraction of TC and NK lineages (Figures 1B and S1C). This reflected a myeloid‐lymphoid imbalance and heightened humoral immunity, potentially indicative of altered differentiation states.

ON‐associated transcriptional signatures were identified using differentially expressed gene (DEG) analysis between all immune cells from the ON and HC groups. We thus defined these genes as ON‐related differentially expressed genes (ON‐DEGs). Upregulated ON‐DEGs included those involved in inflammatory responses (TNF, IL1B, S100A8, S100A9, NLRP3), components of the AP‐1 transcription factor complex (JUNB, FOS, FOSB, JUN), chemokine signaling mediators (CXCL2, CXCL8, CXCR4), and anti‐apoptotic regulators (BCL2A1), whereas those linked to signal transduction adaptors (TRAF2, TIRAP), transcriptional regulation (ID3, NFATC3), and immune inhibition (IL18BP, TNFAIP8) were downregulated (Figure S1E). We then performed gene ontology (GO) analysis to annotate the functions of these genes. Derived pathway enrichment analysis confirmed that leukocyte differentiation, T cell activation, and inflammatory cascades were significantly upregulated in ON (Figure S1F). The downregulated pathways in ON were primarily related to nucleotide metabolism and transportation (Figure S1G).

Next, we performed DEG analysis for each major lineage. Myeloid cells exhibited a pervasive pro‐inflammatory program. Both DC and Mono showed concerted upregulation of key cytokines and chemokines (IL6, TNF, CXCL8, CCL3, CCL4), coupled with induction of stress‐response genes (MAFF, CH25H). In parallel, these cells downregulated genes involved in lipid metabolism (ELOVL1, CD36), immunoregulation (CSF1R, SIGLEC10), and cellular metabolic maintenance (LDHA, SUCLG1), a pattern that may reflect a departure from homeostatic functions. Lymphoid cells showed lineage‐specific alterations in activation, differentiation, and effector function. Among TCs, an activation‐associated profile emerged, marked by the upregulation of the glucose transporter (SLC2A3) and molecules linked to cellular activation and homing (CD69, CXCR4). Conversely, these cells repressed quiescence and immune regulatory functions, as evidenced by reduced CCR7 and IL10RA. Notably, NKs showed a striking downregulation of cytotoxic effector molecules (PRF1, GZMA, GZMM, GNLY) and the activating receptor NCR1, accompanied by the upregulation of transcription factors (ETS1, KLF2) that are frequently associated with stress responses. Indeed, BC primarily displayed evidence of signaling sensitization, characterized by elevated transcription factors and nuclear receptors (FOSB, RORA) concurrent with a corresponding plunge in antigen presentation molecules (CD74). Furthermore, PBC acquired an active survival and differentiation phenotype, upregulating activation markers (CD83, CD69), the pro‐survival kinase AKT1, and immunoglobulin heavy chain variable genes (IGHV family), suggestive of augmented antibody generation. In contrast, cell cycle drivers (MYC, CDK4, AURKB) and B cell adaptor proteins (BLNK, BTK) were otherwise diminished (Figure 1C).

Overall, ON‐DEGs across immune cell subsets were predominantly upregulated, with Mono exhibiting the greatest number of induced genes. Within the lymphoid compartment, TCs showed the strongest transcriptional upregulation, followed by BCs (Figure 1E). Complementary MiloR‐based differential abundance analysis revealed more pronounced ON‐associated compositional changes in TCs than in Mono, with particularly substantial variation across TC subsets (Figure 1F) [26]. Together, these transcriptional and compositional changes motivated our subsequent focus on lymphoid‐cell remodeling. Corroborating this shift, pathway analysis revealed broad enrichment of immune activation and inflammatory signaling, including programs related to T cell activation, lymphocyte differentiation, humoral immunity, and IL‐17 signaling across major immune cell subsets, suggesting a Th17‐skewed inflammatory milieu in ON (Figures 1G and S1H). Indeed, downregulated pathways were largely conserved across cell subsets and were therefore not examined further, as they were primarily enriched for processes related to nucleotide metabolism and protein synthesis, analogous to those observed at the major‐cell level (Figure S1I). Collectively, these findings defined a transcriptional landscape in ON characterized by coordinated inflammatory activation, metabolic reprogramming, and lineage‐specific functional remodeling, with TC‐primed immunity emerging as a major driver.

### 2.2 ON Induction Promotes CD4^+^ T Cell Commitment to CD4^+^ *T*_em_ at the Expense of Treg Cell Differentiation

**FIGURE 2:**
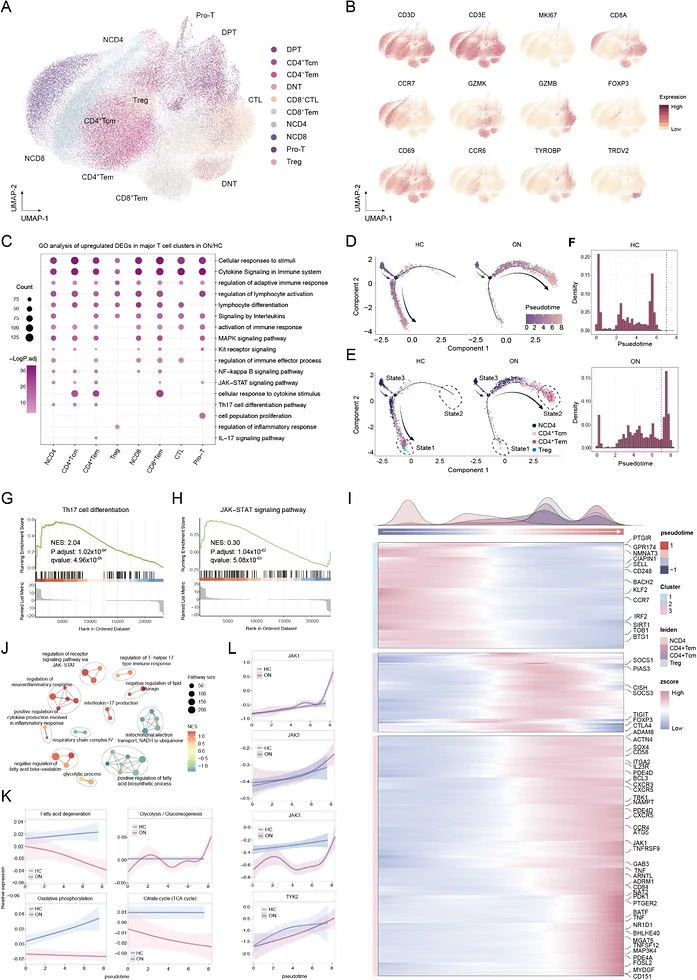
Aberrant differentiation and metabolic reprogramming reshape the CD4^+^ TC repertoire in ON: (A) UMAP plot showing the clustering of major TC subsets from HC and ON samples. (B) UMAP feature plots of canonical TC subset markers. (C) Bubble plot showing representative GO terms enriched in upregulated ON‐DEGs of the TC subsets. (D) Pseudotime trajectory analysis of CD4^+^ TCs, colored by pseudotime. (E) Pseudotime trajectory analysis of CD4^+^ TCs, colored by states and cell types. (F) Histogram showing the cell population distribution corresponding to panels (D,E). (G) GSEA of all expressed genes in TCs revealed significant enrichment of the Th17 differentiation pathway. (H) GSEA of all expressed genes in TCs revealed significant enrichment of the JAK‐STAT signaling pathway. (I) Pseudotime heatmap showing the temporal expression dynamics of key genes along distinct differentiation trajectories in CD4^+^ TC subsets. Rows represent individual genes, and columns represent cells ordered by pseudotime. The top annotation bars indicate pseudotime progression, cluster assignment (Cluster 1–3), and cell subset annotation. Gene expression levels are color‐coded by z‐score. (J) Network plot of KEGG pathway enrichment results derived from GSEA using all genes in CD4^+^ *T*_em_ cells. Each node represents a pathway, node size indicates pathway size, and edges denote pathway‐pathway associations. Node color represents the normalized enrichment score (NES), with color intensity and hue indicating the magnitude and direction (positive or negative) of enrichment. (K) Pseudotime trajectory analysis of key metabolic pathways in CD4^+^ *T*_em_ cells. (L) Expression changes of JAK family genes along the pseudotime trajectory reveal temporal regulation of JAK signaling.

The overall decline in TC frequency led us to speculate that the TC compartment undergoes impaired maintenance and skewed differentiation. To further dissect the distinctive role of TCs in the occurrence of ON, we re‐clustered TCs into 10 major populations, including naïve CD4^+^ T (NCD4) cells, CD4^+^ central memory T (CD4^+^ *T*_cm_) cells, CD4^+^ effector memory T (CD4^+^ *T*_em_) cells, CD4^+^ regulatory T (Treg) cells, naïve CD8^+^ T (NCD8) cells, CD8^+^ effector memory (CD8^+^ *T*_em_) cells, CD8^+^ cytotoxic T cells (CD8^+^ CTL), CD4^−^CD8^−^ T cells (DNT), CD4^+^CD8^+^ T cells (DPT) and proliferative T cells (Pro‐T) (Figure 2A,B and Table S1). Here, we discovered that the naïve pool was preferentially depleted within both conventional CD4^+^ and CD8^+^ TC populations under ON conditions. In CD4^+^ TC, this was accompanied by increased frequencies of CD4^+^ *T*_cm_ and CD4^+^ *T*_em_ cells at the expense of a selective reduction in Treg cells. Similarly, loss of NCD8 was paralleled by an expansion of CD8^+^ CTLs and a decrease in CD8^+^ *T*_em_ cells (Figure S2A). Moreover, our GO analysis results of these TC subsets presented striking enrichment of multiple inflammatory and adaptive immune processes, JAK‐STAT signaling, Th17 differentiation, and the IL‐17 pathway (Figure 2C).

This divergence raised the intriguing possibility that CD4^+^ TC and CD8^+^ TC lineages undergo fundamentally distinct differentiation trajectories in the context of ON. We thus performed pseudo‐time trajectory analysis separately on all CD4^+^ TC and CD8^+^ TC compartments to uncover lineage‐specific fate decisions underlying the observed subset remodeling. For each lineage, the trajectory was rooted in the state most abundant for naïve cells (NCD4 or NCD8 cells), defining the putative starting point. Lineage reconstruction within the CD4^+^ TC compartment revealed distinct fates. In HC, cells predominantly progressed along the branch defining State 1, a cluster enriched for Treg cells. Under ON conditions, however, this homeostatic differentiation route was impaired, with cells preferentially adopting the alternate State 2 branch, which was largely comprised of CD4^+^ *T*_em_ cells (Figure 2D,E). Additionally, CD4^+^ TC density over the differentiation continuum was skewed away from the early peak observed in HC and toward later stages in ON, further mirroring the loss of NCD4 cells and the corresponding rise in CD4^+^ *T*_em_ effectors (Figure 2F). However, the CD8^+^ TC compartment revealed no clear evidence of divergent fate specification in ON. In both HC and ON patients, cells followed a common trajectory without obvious bifurcation into distinct lineage states (Figure S2B,C). Accordingly, pseudotime density profiles remained closely aligned between HC and ON samples, indicative of preserved homeostatic differentiation (Figure S2D). Together, these results suggest that dysregulated CD4^+^ TC differentiation is a key feature of TC remodeling in ON.

### 2.3 JAK1 Emerges as a Candidate Mediator Linking Metabolic Reprogramming to Skewed CD4^+^ T Cell Differentiation in ON

Given the marked redirection of CD4^+^ TC fates toward a CD4^+^ *T*_em_ phenotype, we sought to identify the transcriptional drivers underlying this lineage bias. To this end, we first performed Gene Set Enrichment Analysis (GSEA) on the global TC transcriptome and observed significant enrichment for Th17 differentiation and JAK‐STAT signaling pathways (Figure 2G,H). Pseudotime‐resolved gene clustering delineated three transcriptional modules (Cluster 1–3) that marked sequential and opposing cell‐state programs over the course of CD4^+^ TC differentiation. Cluster 1 corresponded to an early homeostatic/quiescence program (KLF2, SIRT1, BACH2, CCR7), but this module was prematurely extinguished, indicating an early collapse of resting‐state identity. In contrast, Cluster 2 comprised a compensatory inhibitory module enriched for negative regulators of cytokine and TCR signaling (SOCS1, SOCS3, TIGIT, CTLA4). Although its expression increased progressively along pseudotime, the magnitude of induction was blunted, indicating failure to appropriately buffer ongoing activation. Opposing both of these programs, Cluster 3 emerged as the dominant late module and was enriched for expanding proinflammatory effector transcripts, including signaling kinases (JAK1, MAP3K4), transcription factors (BATF, BHLHE40, SOX4), cytokine and chemokine receptors (IL23R, CXCR3, CCR4), and glycolytic metabolic regulators (NAMPT, PDK1). This module continued to intensify with advancing pseudotime, consistent with a progressive shift from homeostatic maintenance, through incomplete inhibitory control, to an effector‐skewed inflammatory and metabolic state (Figure 2I).

Further Kyoto Encyclopedia of Genes and Genomes (KEGG) annotation of the GSEA results revealed a broader metabolic reorganization, wherein pathways associated with glycolysis were enhanced, and those governing oxidative phosphorylation (OXPHOS) and fatty acid catabolism were coordinately diminished (Figure 2J). A division of metabolic labor among CD4^+^ TC subsets has long been implicated in shaping their distinct fates, whereby quiescent naïve TCs rely predominantly on mitochondrial metabolism requiring minimal nutrient uptake, including the tricarboxylic acid (TCA) cycle and OXPHOS, whereas effector TCs adopt glycolysis to fuel rapid energy production [12, 27, 28]. In line with this notion, we discovered that impaired pathways related to mitochondrial respiration were selectively enriched in Treg cells from ON patients (Figure S2E). These findings prompted us to investigate how metabolic programs evolve along the CD4^+^ TC differentiation trajectory.

We next mapped key metabolic gene signatures onto the pseudotime axis. HC CD4^+^ TCs displayed a progressive upregulation of OXPHOS, the TCA cycle, and fatty acid degeneration as they traversed the differentiation continuum. Strikingly, this oxidative trajectory was entirely abolished in ON, where glycolytic activity remained comparatively elevated throughout pseudo‐time (Figure 2K). Notably, JAK‐STAT signaling, a pathway well established to coordinate TC homeostasis, was concomitantly enhanced in our data [29, 30]. We therefore reasoned that its activation could serve as a central hub linking the metabolic and fate alterations observed. Indeed, pseudotime analysis of JAK family kinases revealed that JAK1, but not JAK2, JAK3, or TYK2, was exclusively upregulated at the terminal differentiation stage corresponding to the CD4^+^ *T*_em_‐enriched State 2 (Figure 2L). Furthermore, this pattern was recapitulated in ON CD4^+^ *T*_em_ cells, in which JAK1 was the only JAK family member exhibiting increased expression, while JAK2 remained unchanged and JAK3 and TYK2 were decreased (Figure S2F). Taken together, these results implicated JAK1 as a central integrative node whose activation coincided with metabolic rewiring and excessive CD4^+^ *T*_em_ fate acquisition, thereby potentially licensing the coordinated transition toward a glycolytic, pathogenic state in ON CD4^+^ TCs.

### 2.4 STAT3 Acts as a CD4^+^ *T*_em_‐Specific Transcription Factor That Fuels Pathogenic Differentiation in ON

**FIGURE 3:**
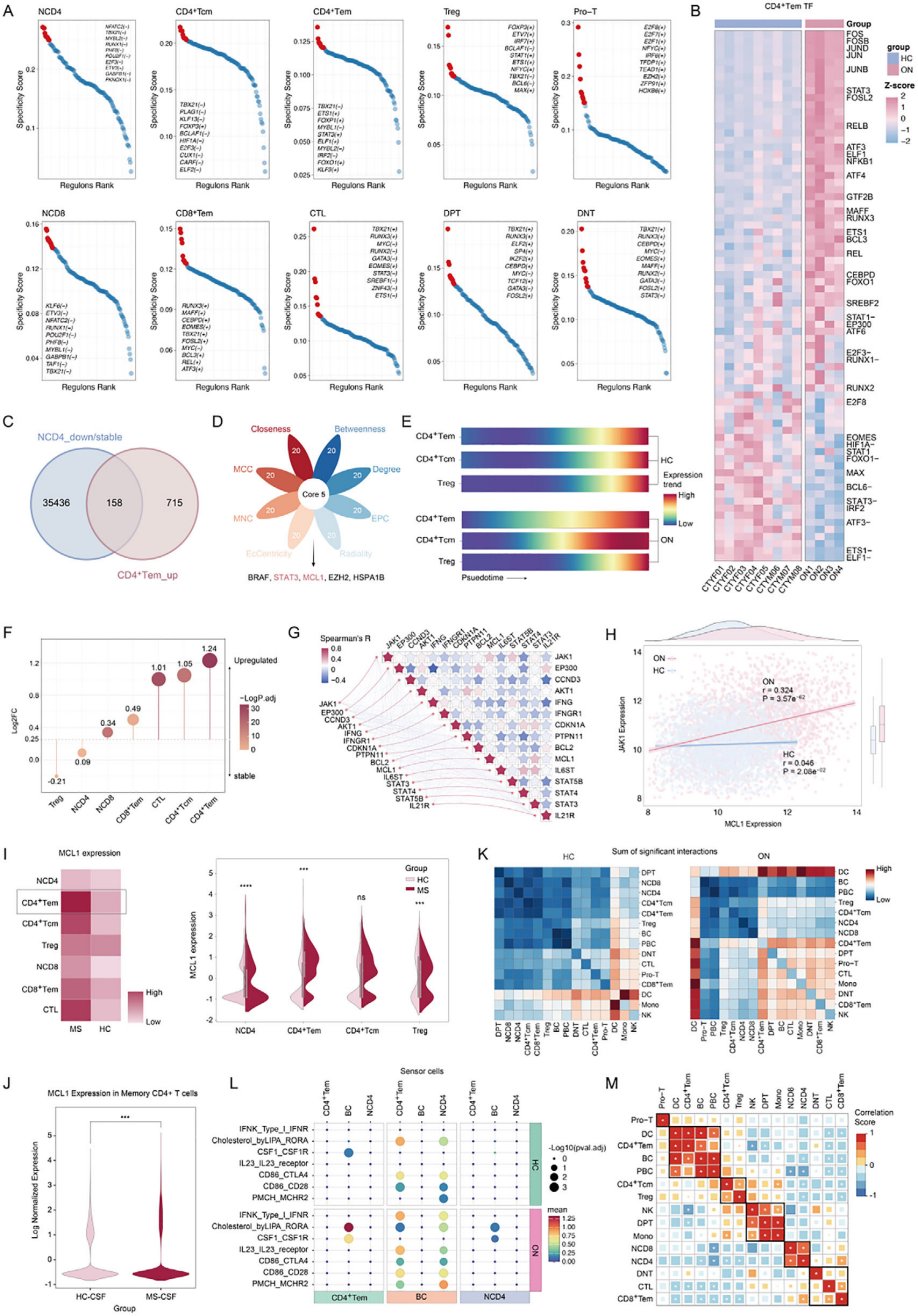
STAT3 is a central transcription factor downstream of JAK1 that drives pathogenic CD4^+^ *T*_em_ differentiation via MCL1 in ON: (A,B) STAT3 regulon is specifically activated in CD4^+^ *T*_em_ cells in ON. (A) SCENIC analysis of all TC subsets showing the ranked regulons based on specificity score; only the top 10 regulons in each subset are displayed and labeled. (B) Heatmap showing the transcription factor/regulon landscape of CD4^+^ *T*_em_ cells across all samples, with scaled activity displayed as Z‐scores. Columns represent individual samples; rows represent transcription factors/regulons. (C–F) MCL1 is identified as a core hub gene specifically upregulated during NCD4‐to‐*T*_em_ differentiation. (C) Venn diagram illustrating the overlap between genes downregulated or stably expressed in NCD4 cells and upregulated ON‐DEGs in CD4^+^ *T*_em_ cells. (D) Venn diagram showing the core hub genes identified from the overlapping genes in (C) using the CytoHubba plugin based on eight algorithms, with MCL1 as a common hit. (E) Pseudotime analysis of MCL1 expression using NCD4 cells as the differentiation root and CD4^+^ *T*_em_, CD4^+^ *T*_cm_, and Treg cells as terminal states, with trajectories displayed separately for HC and ON groups. (F) Lollipop plot showing MCL1 Log_2_ fold change (Log2FC) across TC subsets. (G–J) MCL1 expression correlates with JAK1‐STAT3 signaling and is validated in ON/MS cohorts. (G) Correlation matrix heatmap showing pairwise expression correlations among indicated genes in ON CD4^+^ *T*_em_ cells; color intensity represents the strength of correlation (Spearman's R). (H) Line chart showing the expression correlation between JAK1 and MCL1 in HC versus ON CD4^+^ *T*_em_ cells. (I) Heatmap of average MCL1 expression across TC subsets (left) and violin plots of MCL1 expression in CD4^+^ TC subsets from HC (*n* = 40) and MS patients (*n* = 33) PBMC scRNA‐seq data (right). (J) Violin plots of MCL1 expression in memory CD4^+^ TC subsets from HC (*n* = 13) and MS patients (*n* = 15) CSF scRNA‐seq data. (K–M) CD4^+^ *T*_em_ cells engage in enhanced intercellular communication with B cells in ON. (K) Heatmap showing cellular communication intensity across multiple cell clusters from HC and ON patients. (L) Bubble plot of ligand‐receptor communication showing interactions across different sensor cells, with a focus on NCD4, CD4^+^ *T*_em_, and B cell subsets in HC and ON samples. (M) Pairwise correlation matrix of all immune cell subsets. Significance was evaluated using an unpaired two‐tailed Wilcoxon rank‐sum test. *P < 0.05, **P < 0.01, ***P < 0.001, ****P < 0.0001.

To decipher how JAK1 activation is translated into a lineage‐defining transcriptional program, we next applied SCENIC to ON scRNA‐seq data across all TC subsets to reconstruct gene regulatory networks (GRNs) and infer transcription factor (TF) activity at single‐cell resolution [31]. This analysis revealed distinct regulon architectures across TC subsets. Among them, Treg cells were enriched for canonical regulatory TFs, including FOXP3, IRF4, and STAT1, consistent with an activated immunoregulatory state, while Pro‐T cells preferentially displayed E2F‐driven transcriptional modules, indicative of a proliferative, precursor‐like phenotype. Conversely, cytotoxic and effector‐like populations, including CD8^+^ *T*_em_, CTL, DPT, and DNT cells, were dominated by regulons centered on TBX21, RUNX3, EOMES, CEBPD, and MYC, in line with activated cytotoxic differentiation and inflammatory effector function. Notably, STAT3 emerged as the leading TF in CD4^+^ *T*_em_ cells and was absent from the top regulons of all other TC subsets (Figure 3A). Furthermore, a heatmap was constructed to characterize the transcriptional landscape of CD4^+^ *T*_em_ cells across individual samples. We found that STAT3 activity showed consistent elevation in ON samples compared to HC counterparts in all independent replicates (Figure 3B).

Given that STAT3 is a canonical downstream transcriptional effector of JAK1 signaling and is critically required for the generation of pathogenic Th17 cells, these findings suggested that JAK1 activity may be preferentially transmitted through a STAT3‐driven regulatory network in CD4^+^ *T*_em_ cells during ON [32]. Interestingly, among the TFs profiled, several known regulators of cellular metabolism displayed coordinated alterations in ON CD4^+^ *T*_em_ cells. SREBP2, a master regulator of cholesterol and lipid biosynthesis, exhibited elevated activity relative to HC. Similarly, HIF1A, a key hypoxia sensor that orchestrates glycolytic metabolism, also demonstrated augmented activity in the ON samples (Figure 3B). Such coordinated shifts indicate a state of expansive metabolic reprogramming, wherein CD4^+^ *T*_em_ cells in ON rewired their bioenergetic and biosynthetic infrastructure to meet the demands of chronic inflammatory signaling.

In light of the prominent role of STAT3 in the CD4^+^ *T*_em_ signature and the presumed role of JAK1 as the metabolic switch that licensed pathogenic CD4^+^ *T*_em_ differentiation, we next sought to determine whether STAT3 served as a master regulator of the transcriptional network governing this process. To this end, we performed Venn analysis to intersect the genes downregulated or stably expressed in NCD4 cells (NCD4_down/stable) with those upregulated in CD4^+^ *T*_em_ cells (CD4^+^ *T*_em__up), representing a focused candidate gene set that proactively orchestrates the acquisition of effector memory properties of NCD4 cells. Functional annotation of these candidate genes demonstrated their enrichment in critical immunological pathways, including T cell activation cascades, immune effector modulation, and Th17 lineage commitment (Figure S2G). However, the converse intersection was devoid of these canonical effector programs (Figure S2H,I). To further refine the regulatory hierarchy, we employed the cytoHubba plugin to analyze our protein–protein interaction network constructed from these overlapping genes using eight topological algorithms (MCC, MNC, EcCentricity, Radiality, EPC, degree, Betweenness, and Closeness). Through multi‐algorithm consensus scoring, we robustly prioritized five core hub genes, including BRAF, MCL1, EZH2, HSPA1B, and STAT3, from the top 20 network nodes (Figure 3D). Together, the convergence of STAT3 as both a central hub and a known lineage‐bound TF provided compelling evidence for its role as the master regulator fine‐tuning this pathogenic differentiation program.

### 2.5 MCL1 Acts as an Anti‐Apoptotic Gatekeeper That Supports the Emergence and Maintenance of Pathogenic CD4^+^ *T*_em_ Cells in Autoimmunity

The STAT family represents canonical signal‐dependent TFs that depend on JAK‐dependent phosphorylation for their nuclear translocation and binding to *cis‐*regulatory elements, thereby modulating gene transcription [33]. Mounting evidence has underscored that STATs directly regulate the expression of a wide array of genes, including those involved in cellular metabolism and survival [34, 35, 36, 37, 38]. Given that CD4^+^ TC differentiation requires intricate coordination of metabolic fitness as well as stringent control of apoptosis, we hypothesized that STAT3 might exert its influence by engaging critical regulators of cell survival. In this regard, MCL1 stood out as both a key anti‐apoptotic regulator and a core hub gene that sculpts the CD4^+^ *T*_em_ differentiation landscape (Figure 3D) [24, 39]. Here, we performed palantir analysis to trace MCL1 expression along the separate differentiation trajectories from NCD4 cells toward CD4^+^ memory and regulatory T subsets. In the ON group, an earlier onset of MCL1 upregulation was first detected at the CD4^+^ *T*_em_ stage, while the most pronounced elevation occurred within the CD4^+^ *T*_cm_ compartment. Conversely, the expression pattern in Treg cells remained unchanged (Figures 3E and S2J). Moreover, compared with HC, MCL1 exhibited higher expression levels in both CD4^+^ *T*_em_ and CD4^+^ *T*_cm_ cells, but was downregulated in Treg and remained stable in NCD4 cells (Figure 3F). Strikingly, we also detected a robust and highly coordinated co‐expression pattern encompassing JAK1, STAT3, and MCL1 within the ON CD4^+^ *T*_em_ compartment, and the pairwise relationship between JAK1 and MCL1 was significantly strengthened in the ON group relative to HC (Figure 3G,H). Upon phosphorylation, STAT3 undergoes dimerization and subsequently translocates to the nucleus, where it binds to the MCL1 promoter to activate transcription [40, 41]. MCL1 sequesters pro‐apoptotic effectors BAK/BAX to prevent mitochondrial outer membrane permeabilization and cytochrome c release, thereby blocking caspase activation [42]. Collectively, these findings suggested that a JAK1‐pSTAT3‐MCL1 transcriptional program may sustain the persistence of pathogenic CD4^+^ *T*_em_ cells in the ON inflammatory milieu.

Given the well‐established association between ON and CNS autoimmunity, we next investigated whether the aberrant MCL1 expression signature identified in ON is likewise recapitulated in MS. To this end, we profiled MCL1 expression in CD4^+^ TC subsets isolated from both PBMC and cerebrospinal fluid (CSF) of patients with MS and HC (Figures S3A–F and S4A–C). Consistent with our findings in ON, MCL1 expression in MS patients peaked within the CD4^+^ *T*_em_ compartment, at levels significantly exceeding those in HC, whereas CD4^+^ *T*_cm_ cells showed no appreciable difference. In contrast, both Treg and NCD4 cells exhibited marked downregulation of MCL1 relative to HC (Figure 3I,J). To extend these findings, we queried public transcriptomic datasets for JAK1 and MCL1 expression across a spectrum of autoimmune conditions, including (GCA, Kawasaki disease (KD), primary Sjögren's syndrome (pSS), rheumatoid arthritis (RA), psoriasis (Pso), Crohn's disease (CD), ulcerative colitis (UC), Behçet's disease (BD), systemic lupus erythematosus (SLE), and Vogt–Koyanagi–Harada disease (VKH). Notably, synchronized upregulation of JAK1 and MCL1 within the CD4^+^ *T*_em_ compartment was detected only in GCA and KD (Figure S5A–V). Together, the concerted induction of MCL1 defined a pathogenic CD4^+^ *T*_em_ subset whose footprint radiated across the autoimmune spectrum.

### 2.6 Aberrant Metabolic Crosstalk Between CD4^+^ *T*_em_ Cells and BCs in Optic Neuritis

**FIGURE 4:**
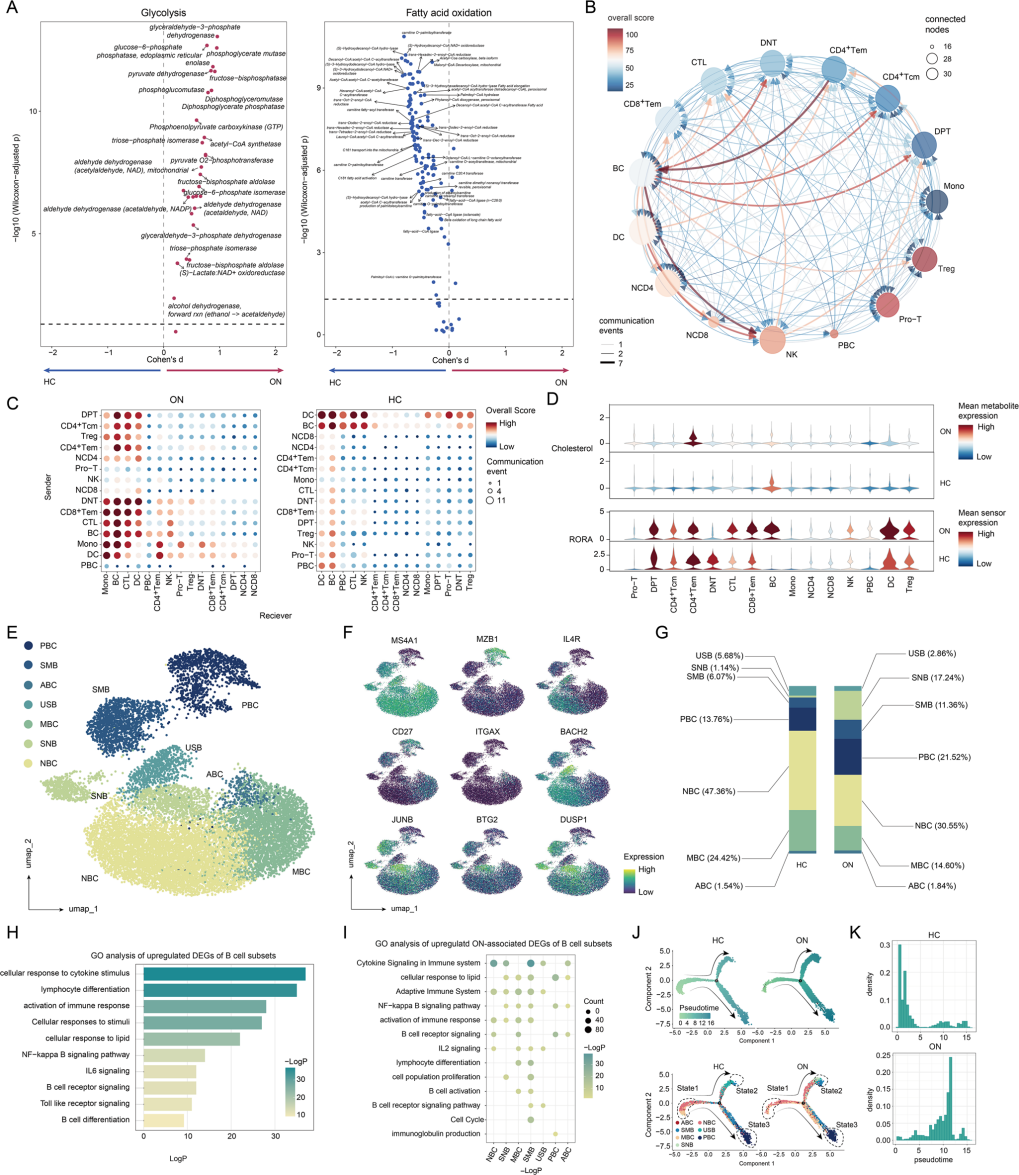
Metabolic reprogramming of CD4^+^ *T*_em_ fuels cholesterol delivery to B cells and drives aberrant autoimmune B cell differentiation in optic neuritis: (A) CD4^+^ *T*_em_ cells undergo glycolytic metabolic reprogramming in ON. COMPASS analysis reveals enhanced glycolysis and suppressed fatty acid oxidation in CD4^+^ *T*_em_ cells from ON patients compared to healthy controls (HC). (B–D) CD4^+^ *T*_em_‐derived metabolites, particularly cholesterol, are unidirectionally transferred to B cells in ON. (B) MEBOCOST analysis identifies unidirectional metabolite transfer from CD4^+^ *T*_em_ to B cells in ON. (C) Comparative heatmap of intercellular metabolite communication among major immune subsets in ON versus HC, highlighting enhanced CD4^+^ *T*_em_‐to‐B cell signaling. (D) Violin plots showing the expression levels of the metabolite cholesterol and its sensor RORA across TC subsets and major immune populations in ON and HC. (E–G) B cell heterogeneity is reshaped in ON. (E) UMAP plot showing the clustering of major B cell subsets from HC and ON samples. (F) UMAP feature plots of canonical B cell subset markers. (G) Bar plots showing the proportion of each B cell subset in HC and ON groups, revealing distinct subset composition changes in ON. (H–J) ON drives functional reprogramming and aberrant differentiation of B cells. (H) Bar plot showing representative GO terms enriched in upregulated DEGs of total B cells in ON. (I) Bubble plot showing representative GO terms enriched in upregulated DEGs across individual B cell subsets. (J) Pseudotime trajectory analysis of B cell subsets in HC and ON samples, indicating skewed differentiation toward pathogenic terminal states in ON. (K) Histogram showing the cell population distribution corresponding to panel J. Significance was evaluated using an unpaired two‐tailed Student's *t*‐test. *P < 0.05, **P < 0.01, ***P < 0.001, ****P < 0.0001.

While effective immunity hinges on intricate intercellular communication among immune subsets, it remains unclear whether the prominently expanded and effector‐skewed CD4^+^ *T*_em_ cells in ON actively reconstruct this broader communication network. To address this, we inferred ligand–receptor interactions between TC subsets and other major immune cell populations in ON and HC samples. The intercellular communication landscape was extensively remodeled in ON, with a global upregulation of inferred ligand–receptor interactions. Notably, this remodeling prominently featured enhanced reciprocal crosstalk between DCs and multiple TC subsets, including CD4^+^ *T*_em_ cells (Figure 3K). Among the reinforced DC‐TC interactions, pathways involving CCL chemokines, LCK, CD86, and SEMA4 family members were particularly elevated, implicating a reconstructed communication landscape in ON patients (Figure S6E–H). Intriguingly, we observed strengthened communication between BCs and CD4^+^ *T*_em_ cells. Indeed, the IL‐23/IL‐23R axis, a key driver of Th17 differentiation and survival, was virtually undetectable in HC yet markedly elevated in ON. In addition, co‐stimulatory signaling at the TC‐BC interface shifted from the inhibitory CD86‐CTLA4 axis in HC to the activating CD86‐CD28 axis in ON. Specifically, CD4^+^ *T*_em_ cells engaged BCs through a cholesterol‐RORA ligand–receptor pair, hinting at an additional layer of metabolic crosstalk (Figure 3L). Moreover, CD4^+^ *T*_em_ cells, BCs, and PBCs were highly interconnected in ON (Figure 3M). This raises the possibility that metabolic rewiring in CD4^+^ *T*_em_ cells enabled them to instruct the behavior of bystander immune cells, thereby perpetuating autoimmunity. Thus, we first sought to define the intrinsic metabolic configuration of CD4^+^ *T*_em_ cells affected by ON. Compass analysis revealed that ON CD4^+^ *T*_em_ cells exhibit a metabolic configuration marked by heightened glycolytic flux and compromised OXPHOS. This was evidenced by enhanced glycolysis, fructose‐mannose metabolism, and fatty acid synthesis, coupled with impaired FAO and diminished TCA cycle as well as cholesterol metabolism (Figures 4A and S6A–D).

We next applied MEBOCOST to infer metabolite‐mediated communication between CD4^+^ *T*_em_ cells and other immune subsets in ON and HC. Global metabolite‐sensor interactions were broadly enhanced in ON relative to HC. Notably, this enhanced communication was exclusively directed from CD4^+^ *T*_em_ cells toward BCs in ON (Figures 4B,C and S6I). Consistently, CD4^+^ *T*_em_ cells in ON displayed increased expression of SLC2A3 and PTGER4, and decreased S1PR1, delineating a metabolically active, pro‐inflammatory, and tissue‐resident effector state (Figure S6J). Delving deeper, CD4^+^ *T*_em_ cells in ON also manifested elevated expression of cholesterol efflux transporters. Meanwhile, cholesterol sensor RORA, though broadly expressed across immune subsets, was solely stimulated in BCs (Figure 4D). This divergent pattern suggested that cholesterol metabolites exported from CD4^+^ *T*_em_ cells may engage RORA in BCs during ON induction.

### 2.7 Inflammatory B and PBC Expansion at the Expense of Diminished Regulatory Subsets During ON Progression

Dysregulated cholesterol metabolism is intimately linked to aberrant BC activation and autoantibody production [43, 44]. In ON, BCs exhibited broad enrichment for pathways involved in IL‐6 production, IL‐12 production, and MHC class II assembly, indicative of a global shift toward a pro‐inflammatory phenotype. Correspondingly, FAO‐associated pathways were markedly activated, while cholesterol efflux signatures were diminished, suggesting that BC activation and differentiation in ON are accompanied by heightened dependence on cholesterol availability (Figure S6K). This finding further consolidated our speculation that CD4^+^ *T*_em_‐derived cholesterol metabolites may fan the flames of BC autoimmunity in ON.

We therefore asked whether BCs undergo a characteristic shift in differentiation and functional polarization during ON induction. BCs were then classified into 7 clusters: Age‐related B (ABC) cell, unswitched B (USB) cell, Memory B cell (MBC), naïve B cell (NBC), Stimulated Memory B (SMB) cell, SNB (Stimulated naïve B) cell, and PBC (Figure 4E,F and Table S1). In ON, naïve and resting memory BC subsets (NBC, MBC, USB) were uniformly reduced, whereas stimulated subsets (SNB, SMB) and PBC were expanded (Figure 4G). GO analysis highlighted a global transcriptional signature of inflammatory activation across the BC compartment in ON (Figure 4H). Intriguingly, SMB, PBC, and MBC were the only BC subsets that shared both lipid‐responsive pathway enrichment and JAK1 upregulation in ON (Figure S6L). This convergence implied that lipid sensing and JAK1 activity may act in concert to confer pathogenic properties upon memory and autoreactive BCs in ON. Notably, ABCs displayed a selective defect in IL‐2 signaling (Figure 4I). Previous studies have shown that BC‐intrinsic IL‐2 signaling promotes the MAF‐dependent differentiation of CD25^+^ ABCs into IL‐10‐producing regulatory cells that restrain neuroinflammation [45]. The selective defect in IL‐2 signaling observed in ON ABCs therefore indicates a loss of this protective immunoregulatory capacity. Furthermore, a loss of NBCs and MBCs at the trajectory origin led to a substantial depletion of the quiescent differentiation state (State 1) in ON. Thereafter, the path bifurcated into two distinct branches. One branch progressed toward a terminal inflammatory fate and was predominantly populated by SNBs and SMBs (State 2), while the other was enriched for PBCs, as MBCs and SMBs both exhibited a strong directional bias toward PBC differentiation along this path (State 3). These trajectory deviations were specific to ON and were absent in HC BCs (Figure 4J,K). Density plots along the pseudotime revealed that HC BCs were predominantly concentrated in the quiescent state (State 1), whereas ON BCs were characterized by a broad redistribution toward the activated branches (States 2 and 3), reflecting a global shift from quiescence to differentiation and inflammatory polarization.

Collectively, these findings implicated a pathological interplay in ON wherein metabolically primed CD4^+^ *T*_em_ cells furnished cholesterol metabolites that were hijacked by the BC sensor RORA to commandeer the gain of BC autoreactivity. This metabolic interception precipitated the erosion of quiescent and regulatory fidelities and the unbridled expansion of pro‐inflammatory and PBC‐committed clones. In a reciprocal fashion, these subverted BCs may provide co‐stimulatory and cytokine‐mediated cues that further reinforced the differentiation of pathogenic CD4^+^ *T*_em_ cells, thereby entrenching a self‐perpetuating cycle of neuroinflammation.

### 2.8 Upadacitinib Attenuates Disease Progression and Immune Dysregulation in EAE Mice

The observations that JAK1 signaling was selectively heightened in pathogenic CD4^+^ *T*_em_ cells and concurrently upregulated in the SMB, PBC, and MBC subsets of BCs suggested that JAK1 may serve as a shared molecular pivot that coordinates the reciprocal reprogramming of TC and BC compartments in ON. Given this dual involvement, pharmacological JAK1 blockade could, in principle, simultaneously interrupt TC‐intrinsic reprogramming, as well as the cholesterol‐RORA conduit from TCs to BCs. Upadacitinib (UPA), an oral, reversible JAK1‐selective inhibitor, has demonstrated robust efficacy and an acceptable safety profile in several autoimmune diseases. However, whether UPA confers therapeutic benefit in neuroinflammatory disorders, particularly ON, remains elusive.

**FIGURE 5:**
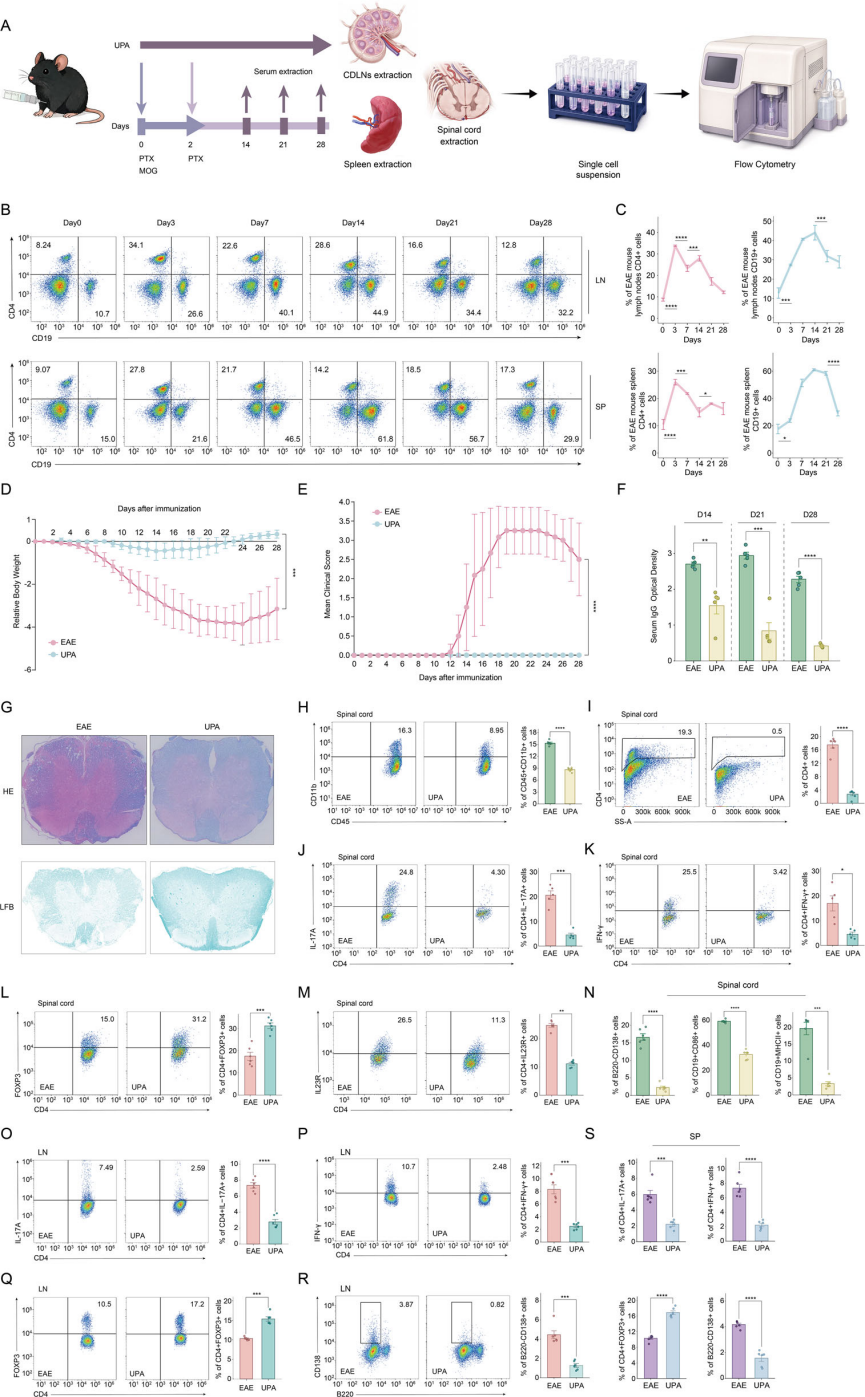
Upadacitinib attenuates clinical disease progression and neuroinflammation in EAE: (A) Experimental design. Schematic workflow of EAE induction, UPA treatment, and sample collection for flow cytometry and histopathological analysis. (B,C) Dynamic changes of CD4^+^ T and CD19^+^ B cells during EAE progression. (B) Representative flow cytometry plots and (C) line graphs showing the proportions of CD4^+^ TCs and CD19^+^ BCs in lymph nodes (LNs) and spleen (SP) at consecutive days after EAE induction (*n* = 6 per time point). (D–G) UPA ameliorates clinical severity and neuropathology in EAE. (D) Relative body weight changes and (E) daily clinical scores in EAE versus UPA‐treated mice (*n* = 5 per group). (F) Serum IgG ELISA signals at days 14, 21, and 28 post‐immunization (*n* = 5 per group). (G) H&E and LFB staining of spinal cord sections showing reduced inflammatory infiltration and demyelination upon UPA treatment. (H–N) UPA reduces pathogenic T and B cell responses in the spinal cord. Flow cytometric analysis of spinal cord infiltrates from EAE and UPA mice, including (H) CD45^+^CD11b^+^ myeloid cells, (I) total CD4^+^ TCs, (J) CD4^+^IL‐17A^+^ Th17 cells, (K) CD4^+^IFN‐γ^+^ Th1 cells, (L) CD4^+^FOXP3^+^ Treg cells, (M) CD4^+^IL‐23R^+^ TCs, and (N) B220^−^CD138^+^ plasma cells (PBCs), CD19^+^CD86^+^ B cells, and CD19^+^MHCII^+^ B cells (*n* = 6 per group). (O–R) UPA modulates T and B cell phenotypes in peripheral lymph nodes. (O–Q) Expression of IL‐17A, IFN‐γ, and FOXP3 in CD4^+^ TCs from LNs of EAE and UPA mice (*n* = 5 per group). (R) Proportion of B220^−^CD138^+^ PBCs in LNs (*n* = 5 per group). (S) UPA modulates T and B cell phenotypes in the spleen. Expression of IL‐17A, IFN‐γ, and FOXP3 in CD4^+^ TCs and B220^−^CD138^+^ PBCs from SP of EAE and UPA mice (*n* = 5 per group). Data are presented as mean ± SD. Statistical differences for daily clinical scores and relative body weight were analyzed by two‐way ANOVA. All other statistical analyses were performed using unpaired two‐tailed Student's *t*‐test. *p < 0.05, **p < 0.01, ***p < 0.001, ****p < 0.0001; ns, not significant.

Therefore, we next evaluated the effects of UPA in experimental autoimmune encephalomyelitis (EAE), a murine model that mirrors the optic nerve inflammation and demyelination characteristic of ON [8]. We established the EAE model by immunizing 6–8 weeks old C57BL/6J mice with myelin oligodendrocyte glycoprotein peptide 35–55 (MOG_35‐55_) emulsified in complete Freund's adjuvant (CFA), followed by intraperitoneal administration of pertussis toxin (PTX). UPA (10 mg/kg, qd, i.p.) or vehicle was administered from the day of EAE induction and continued daily for 28 days (Figure 5A). Longitudinal profiling revealed a biphasic CD4^+^ TC response characterized by an early priming‐associated expansion, a subsequent decline coinciding with CD19^+^ BC expansion, and a later rebound consistent with a reciprocal TC‐BC loop (Figure 5B,C). UPA markedly reduced clinical scores and protected against weight loss (Figure 5D,E). Serum IgG signals were lower at all measured time points (days 14, 21, and 28), while H&E and LFB staining showed less inflammatory infiltration and demyelination in the spinal cord (Figure 5F,G). Flow cytometry further showed lower frequencies of CD45^+^CD11b^+^ myeloid cells, total CD4^+^ TCs, Th1 and Th17 cells, CD4^+^IL‐23R^+^ TCs, PBCs, and activated CD86^+^, MHCII^+^, CD69^+^ BCs, together with a higher proportion of Tregs (Figures 5H–N and S8A–D). The gating strategy is shown in Figure S7.

**FIGURE 6:**
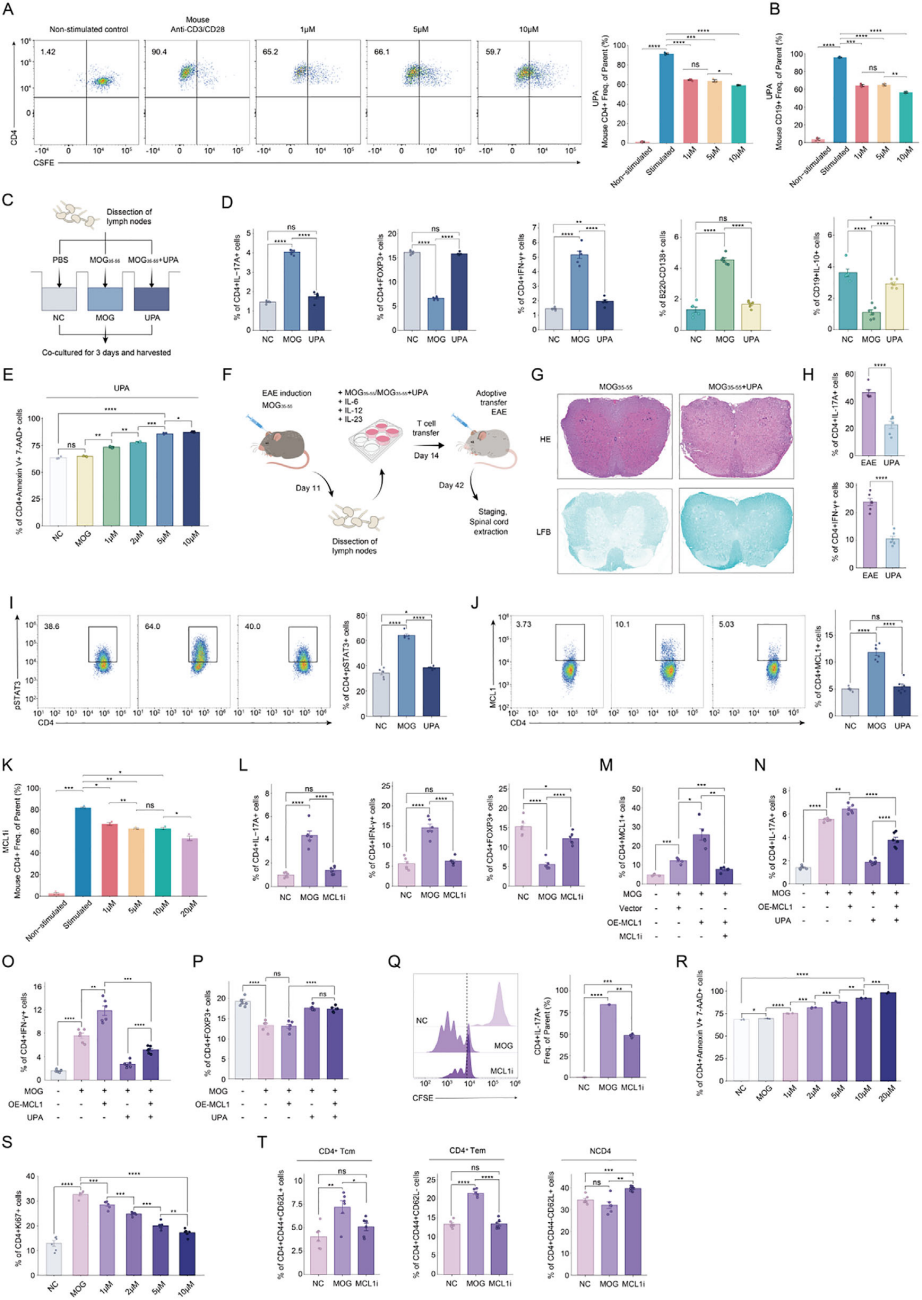
Upadacitinib rebalances TC responses and modulates BC compartments in EAE by suppressing pSTAT3‐MCL1 signaling in CD4^+^ TCs: (A–E) Upadacitinib (UPA) modulates pathogenic T/B cell responses in vitro. (A,B) CFSE dilution assays showing the effects of UPA at indicated concentrations on the proliferation of mouse CD4^+^ T cells (A) and CD19^+^ B cells (B) (*n* = 3 per group). (C) Schematic workflow of in vitro cell culture and treatment. (D) Flow cytometric analysis of IL‐17A, IFN‐γ, and FOXP3 in CD4^+^ T cells, IL‐10 in CD19^+^ B cells, and B220^−^CD138^+^ plasma cells (PBCs) under NC, MOG_35‐55_ (MOG), and MOG plus UPA conditions (*n* = 6 per group). (E) Annexin V/7‐AAD profiles assessed across NC, MOG, and MOG plus gradient‐dose UPA groups (*n* = 3 per group). (F–H) UPA‐pretreated CD4^+^ T cells attenuate EAE pathology in an adoptive‐transfer model. (F) Schematic of the adoptive‐transfer EAE model: MOG‐primed CD4^+^ T cells were treated with or without UPA in vitro and transferred into recipient mice. (G) H&E and LFB staining of spinal cord sections from recipient mice. (H) Proportions of CD4^+^IL‐17A^+^ Th17 and CD4^+^IFN‐γ^+^ Th1 cells in the spinal cord (*n* = 6 per group). (I–P) MCL1 modulates pathogenic T cell phenotypes. (I,J) Frequencies of CD4^+^pSTAT3^+^ (I) and CD4^+^MCL1^+^ (J) T cells under NC, MOG, and UPA conditions (*n* = 6 per group). (K) CFSE dilution assay of mouse CD4^+^ T cells treated with gradient concentrations of MCL1 inhibitor (MCL1i) (*n* = 3 per group). (L) IL‐17A, IFN‐γ, and FOXP3 expression in CD4^+^ T cells under NC, MOG, and MOG plus MCL1i conditions (*n* = 6 per group). (M) CD4^+^MCL1^+^ T cell frequencies under NC, MOG plus vector, MOG plus OE‐MCL1, and MOG plus MCL1i plus OE‐MCL1 conditions (*n* = 5 per group). (N–P) Proportions of Th17 (N), Th1 (O), and Treg (P) cells under NC, MOG, MOG plus OE‐MCL1, MOG plus UPA, and MOG plus OE‐MCL1 plus UPA conditions (*n* = 6 per group). (Q–T) MCL1 modulates T cell proliferation, Annexin V/7‐AAD profiles, and memory‐phenotype composition. (Q) CFSE dilution assay of CD4^+^IL‐17A^+^ Th17 cells treated with MCL1i (*n* = 3 per group). (R) Annexin V/7‐AAD profiles assessed across NC, MOG, and MOG plus gradient‐dose MCL1i groups (*n* = 3 per group). (S) Frequencies of CD4^+^Ki67^+^ proliferating T cells under NC, MOG, and MOG plus MCL1i (*n* = 6 per group). (T) Proportions of CD4^+^ naive (NCD4), central memory (*T*_cm_), and effector memory (*T*_em_) T cells under the indicated conditions (*n* = 6 per group). Data are shown as mean ± SD. Significance was evaluated using unpaired two‐tailed Student's *t*‐test: *P < 0.05, **P < 0.01, ***P < 0.001, ****P < 0.0001, and ns for not significant.

The peripheral response was rebalanced in parallel. UPA treatment also rebalanced the systemic immune landscape in EAE mice. Evaluation of lymph nodes and spleens revealed that UPA greatly dampened the proportions of pathogenic Th1, Th17, and mature PBC while concomitantly augmenting the frequency of protective Treg cells (Figures 5O–S and S8E–G). To further explore the function of UPA in EAE, in vitro experiments were conducted using cells isolated from EAE‐induced mice. Cells were restimulated with MOG_35‐55_ in the presence of UPA or vehicle, with unstimulated cells serving as blank controls (Figure 6C). UPA at 5 µm had no impact on cell viability but effectively depressed the proliferation of CD4^+^ TCs and CD19^+^ BCs (Figures 6A,B and S9F). Therefore, we chose this dose for the following experiments. Notably, UPA treatment decreased the proportions of Th1, Th17, and mature PBCs while expanding the Treg compartment (Figures 6D and S9A–D). Intriguingly, UPA treatment also substantially restored the frequency of IL‐10‐producing regulatory B (Breg) cells, a subset known to restrain disease activity and progression in ON and other autoimmune disorders [13] (Figures 6D and S9E). Thus, pharmacological inhibition of JAK1 with UPA appeared to partially reinstate the immunoregulatory capacity of BC, counteracting the pathogenic skewing observed in ON and EAE. In an adoptive‐transfer model, MOG_35‐55_‐primed CD4^+^ TCs were exposed to UPA before transfer into recipient mice. Recipients developed milder clinical disease and less weight loss, findings that were paralleled by reduced spinal‐cord inflammation, demyelination, and lower frequencies of Th1 and Th17 cells in the spinal cord (Figures 6F–H and S9H–K). These adoptive‐transfer findings complement systemic treatment studies by showing that UPA‐exposed CD4^+^ TCs are sufficient to contribute to the observed protection.

Collectively, these findings established UPA as a potent therapeutic agent that resolved EAE pathology by concurrently extinguishing pathogenic lymphocyte responses and fortifying regulatory TC and BC compartments.

### 2.9 JAK1‐pSTAT3‐MCL1 Signaling Sustains Pathogenic CD4^+^ TC Fitness and the Effector‐Memory Phenotype in EAE

The robust therapeutic activity of UPA in EAE amelioration prompted us to interrogate the underlying mechanism. Our prior discovery in ON defined a JAK1‐pSTAT3‐MCL1 signaling axis that is selectively heightened in pathogenic CD4^+^ *T*_em_ cells and confers both differentiation competence and survival advantage. We therefore reasoned that UPA exerts its protective effects in EAE by dismantling this pathogenic cascade. Indeed, MOG_35‐55_ stimulation increased pSTAT3 and MCL1 in CD4^+^ TCs, and UPA reversed both changes (Figure 6I,J). Recapitulating the immunomodulatory signature of UPA, the MCL1 inhibitor A‐1210477 (MCL1i) profoundly suppressed Th1 and Th17 polarization while reinstating the Treg compartment in vitro (Figure 6K,L,Q,S; Figures S9L and S10A–C,I).

In MOG_35‐55_‐stimulated cultures, MCL1 overexpression increased the frequency of CD4^+^MCL1^+^ TCs compared with the vector control, an effect that was abrogated by MCL1i (Figure 6M). MCL1 overexpression also partially opposed the suppression of Th17 and Th1 cells by UPA (Figures 6N,O and S10E,F), whereas it did not appreciably reverse UPA‐associated Treg recovery (Figure 6P). MCL1 inhibition further reduced CD4^+^Ki67^+^ TCs and shifted the CD4^+^ compartment away from *T*_em_/*T*_cm_ states toward the NCD4 phenotype (Figures 6S,T and S10I). Together, these data cemented MCL1 as the pivotal downstream conduit of JAK1‐pSTAT3 signaling in EAE and validated its blockade as a mechanistic surrogate for UPA in suppressing pathogenic CD4^+^ TC responses.

### 2.10 JAK1‐pSTAT3‐Driven TC Glycolysis Promotes CD4^+^ Tem Differentiation and Cholesterol Efflux‐Dependent PBC Generation

**FIGURE 7:**
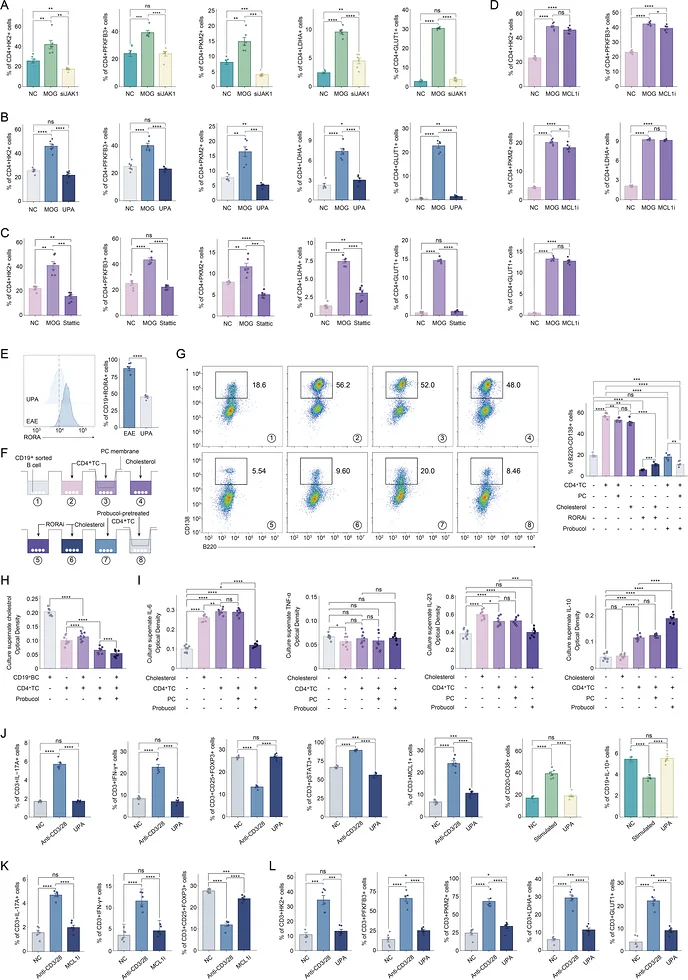
JAK1‐pSTAT3 signaling regulates glycolysis‐related proteins in activated CD4^+^ TC and is associated with cholesterol‐sensitive modulation of BC responses in optic neuritis: (A–D) JAK1‐pSTAT3 axis regulates glycolysis‐related protein expression in CD4^+^ T cells under MOG_35‐55_ stimulation. Flow cytometric analysis of HK2, PFKFB3, PKM2, LDHA, and GLUT1 in CD4^+^ T cells. Cells were divided into non‐stimulated control (NC), MOG_35‐55_ (MOG), and MOG plus intervention groups: (A) JAK1 siRNA (siJAK1), (B) upadacitinib (UPA), (C) STAT3 inhibitor (Stattic), and (D) MCL1 inhibitor (MCL1i) (*n *= 6 per group). (E) UPA treatment reduces CD19^+^RORA^+^ B cells in EAE mice. Left: representative histogram of RORA expression in EAE (light blue) versus UPA‐treated (dark blue) groups. Right: quantification of CD19^+^RORA^+^ cell percentage (*n* = 6 per group). (F) Schematic of the in vitro co‐culture system for CD19^+^ B cells and CD4^+^ T cells. Experimental conditions: ① Sorted CD19^+^ B cells (baseline control). ② CD4^+^ T cells added to CD19^+^ B cell culture. ③ CD4^+^ T cells co‐cultured with CD19^+^ B cells in a transwell chamber separated by a polycarbonate (PC) membrane. ④ Cholesterol supplementation to CD19^+^ B cell culture. ⑤ RORA inhibitor (RORAi) treatment of CD19^+^ B cells. ⑥ Cholesterol treatment of RORAi‐pretreated CD19^+^ B cells. ⑦ Probucol‐pretreated CD4^+^ T cells added to CD19^+^ B cell culture. ⑧ Probucol‐pretreated CD4^+^ T cells co‐cultured with CD19^+^ B cells in a transwell chamber separated by a PC membrane. (G) Co‐culture perturbations support a RORA‐dependent, cholesterol‐sensitive route associated with PBC differentiation. Representative flow cytometry plots and quantification of B220^−^CD138^+^ PBCs under the co‐culture conditions described in (F). Bar graphs show statistical summary across all groups (*n* = 6 per group). (H,I) Probucol alters cholesterol‐assay signals and cytokine secretion in co‐culture supernatants. (H) Cholesterol‐assay optical‐density (OD) signals across groups with or without B cells, T cells, and probucol treatment (*n *= 8 per group). (I) ELISA quantification of pro‐inflammatory (IL‐6, TNF‐α, IL‐23) and anti‐inflammatory (IL‐10) cytokines across experimental groups (*n* = 8 per group). (J–L) Validation of UPA and MCL1i effects on T/B cell subsets and T cell glycolysis in ON patient PBMCs. (J) PBMCs from ON patients were stimulated with anti‐CD3/CD28 or CpG ODN1826/CD40L/IL‐4/IL‐21 with or without UPA, followed by flow cytometric analysis of Th17 (CD3^+^CD8^−^IL‐17A^+^), Th1 (CD3^+^CD8^−^IFN‐γ^+^), Treg (CD3^+^CD8^−^CD25^+^FOXP3^+^), CD3^+^CD8^−^pSTAT3^+^ T cells, CD3^+^CD8^−^MCL1^+^ T cells, CD20^−^CD38^+^ PBCs, and CD19^+^IL‐10^+^ regulatory B cells (Breg) (*n* = 6 per group). (K) Proportions of Th17, Th1, and Treg cells in PBMCs treated with anti‐CD3/CD28 plus MCL1i (*n* = 6 per group). (L) Flow cytometric analysis of glycolysis‐related proteins (HK2, PFKFB3, PKM2, LDHA, and GLUT1) in human CD4^+^ T cells under NC, anti‐CD3/CD28, or anti‐CD3/CD28 plus UPA conditions (*n* = 6 per group). Data are shown as mean ± SD. Significance was evaluated using an unpaired two‐tailed Student's *t*‐test. *P < 0.05, **P < 0.01, ***P < 0.001, ****P < 0.0001, and ns for not significant.

To determine whether JAK1‐pSTAT3‐MCL1 signaling controlled the glycolytic phenotype predicted by the single‐cell data, we measured hexokinase 2 (HK2), 6‐phosphofructo‐2‐kinase/fructose‐2,6‐bisphosphatase 3 (PFKFB3), pyruvate kinase M2 (PKM2), lactate dehydrogenase A (LDHA), and glucose transporter 1 (GLUT1) in MOG_35‐55_‐stimulated CD4^+^ TCs. Genetic silencing of JAK1, or pharmacological inhibition of JAK1 with UPA or STAT3 with Stattic, broadly suppressed the upregulation of these proteins, whereas MCL1i did not exert comparable broad metabolic suppression (Figures 7A–D and S11). These findings distinguish a JAK1‐pSTAT3 metabolic axis from the predominantly fitness‐related functions of MCL1.

Expectedly, UPA also reduced CD19^+^RORA^+^ BCs in EAE mice (Figure 7E). In transwell co‐cultures, CD4^+^ TCs promoted PBC differentiation even when physically separated from BCs, and exogenous cholesterol elicited a similar response, suggesting a cholesterol‐mediated, contact‐independent mechanism. Accordingly, treatment of BCs with a RORA inverse agonist (RORAi) or pretreatment of TCs with the cholesterol‐efflux inhibitor probucol markedly suppressed PBC generation (Figure 7F,G). CFSE dose‐response assays informed selection of Stattic, RORAi, and probucol concentrations (Figure S12A–C). Probucol reduced supernatant cholesterol levels, decreased IL‐6 and IL‐23, and increased IL‐10, whereas TNF‐α was largely unchanged (Figure 7H,I). Collectively, these data support a cholesterol‐sensitive, contact‐independent route by which TCs modulate RORA‐associated BC differentiation.

### 2.11 UPA Reverses Pathogenic Immune and Glycolytic Programs in Human ON PBMCs In Vitro

To extend the findings from the EAE mouse model to human neuroinflammatory disease, we investigated the role of the JAK1‐pSTAT3‐MCL1 axis in modulating immune cell dynamics in PBMCs derived from patients with ON. UPA at 5 µm did not affect cell viability yet markedly curtailed the proliferation of both human CD4^+^ TCs and CD19^+^ BCs upon stimulation (Figure S12D,E). Anti‐CD3/CD28 stimulation elicited a pro‐inflammatory shift in ON‐derived PBMCs, characterized by elevated Th1/Th17 frequencies and a concomitant collapse in Treg proportions. However, UPA treatment potently extinguished Th1 and Th17 frequencies and replenished Treg proportions diminished by anti‐CD3/CD28 stimulation. Stimulation of PBMCs with CpG ODN1826, CD40L, IL‐4, and IL‐21 efficiently induced the generation of mature PBCs and handicapped IL‐10‐producing Breg, an effect that was profoundly offset by UPA treatment (Figure 7J; Figures S12G–L and S13A). In addition, UPA broadly reduced the expression of HK2, PFKFB3, PKM2, LDHA, and GLUT1 in CD4^+^ TCs (Figure 7L; Figures S13E–I). Similarly, MCL1i at 10 µm spared cell viability but significantly suppressed CD4^+^ TC expansion and recapitulated the immunomodulatory profile of UPA in stimulated ON PBMCs, as evidenced by diminished Th1/Th17 frequencies and a concomitant recovery of the Treg compartment (Figure 7K; Figures S12F and S13B–D).

In conclusion, these findings affirmed the conserved function of UPA in recalibrating the Th1/Th17 and Treg cell imbalance through targeted disruption of the JAK1‐pSTAT3‐MCL1 cascade in both ON patients and EAE mice. UPA thus emerged as a compelling therapeutic candidate for reinstating immune homeostasis in ON and related autoimmune disorders.

## 3 Discussion

Our study introduced a pathological framework that positions the JAK1‐STAT3 transcriptional cascade as a master coordinator of CD4^+^ *T*_em_ cell fitness and BC dysregulation underlying ON autoimmunity. At the heart of this signaling modality lay a transcriptional module that coupled metabolic rewiring with imputed cholesterol export activity in pathogenic CD4^+^ *T*_em_ cells, instructing bystander BCs toward an autoreactive fate. In turn, heightened antigen presentation and pro‐inflammatory cytokine programs in BCs could reciprocally consolidate pathogenic CD4^+^ *T*_em_ cell persistence, suggesting a propensity for a self‐amplifying inflammatory circuit. Notably, MCL1 emerged as a core hub within this network. Pharmacological blockade of JAK1 with UPA reversed the JAK1‐pSTAT3‐MCL1 signaling cascade in CD4^+^ TCs and aligns with attenuated BC pathogenic responses, thereby contributing to the restoration of immune homeostasis in both compartments. Our findings nominated JAK1 as a shared molecular pivot coordinating cross‐lineage autoreactive lymphocyte responses, and identified MCL1 as its downstream executor of CD4^+^ *T*_em_ cell fitness. This dual action, which we termed “bilateral immune resetting”, thus offered a mechanistically grounded therapeutic strategy for ON and potentially other neuroinflammatory disorders driven by coordinated TC‐BC dysregulation.

Canonically, JAK1 is perceived as a passive conduit that links extracellular cues, such as IL‐6 or type I interferons, to STAT‐dependent transcriptional outputs, thereby influencing TC lineage commitment [20]. In addition, the JAK‐STAT pathway has long been implicated in metabolic control in activated TCs. Notably, our transcriptomic profiling brought the JAK1‐STAT3 signaling axis to the forefront in pathogenic CD4^+^ *T*_em_ cells in ON. This observation takes on added significance in light of evidence that Stat3 can directly bind the HIF‐1α promoter to drive its transcription in mice, thereby enforcing glycolytic metabolism to tip the Th17/Treg cell balance [46]. In agreement with this, our TF activity inference identified HIF1A as a core regulon with elevated activity in pathogenic CD4^+^ *T*_em_ cells. Our functional perturbation data now show that JAK1‐STAT3 blockade broadly suppresses glycolysis‐related proteins in MOG_35‐55_‐stimulated mouse CD4^+^ TCs and stimulated human CD4^+^ TCs, linking the inferred transcriptional program to metabolic function. These concordant mouse and human in vitro effects connect the inferred transcriptional state to a measurable metabolic phenotype, while stopping short of resolving transcriptional versus post‐transcriptional control. Intriguingly, a recent study has shown that the polyamine spermine directly binds and inhibits JAK1 to restrain cytokine‐driven autoimmunity, illustrating that metabolic cues can, in turn, gatekeep JAK1 activity [47]. Furthermore, STAT3 activity itself is subject to regulation by a lipid metabolite through a reversible cycle of S‐palmitoylation, a post‐translational modification involving the covalent attachment of the fatty acid palmitate that enhances STAT3 activation and promotes Th17 differentiation [48]. Collectively, these findings reframe the JAK1‐STAT3 cascade as a node that integrates both cytokine and metabolic inputs. Rather than a passive conduit, JAK1‐STAT3 signaling may therefore function as a rheostat that tunes the intrinsic fitness of pathogenic CD4^+^ *T*_em_ cells. Accordingly, resetting this rheostat may therefore represent a targeted approach for autoimmune diseases fueled by aberrant TC fitness.

The temporal and co‐culture data together suggest a staged, reciprocal interaction between TCs and BCs. In EAE, CD4^+^ TCs expanded early after immunization, consistent with initial priming, then declined as BCs accumulated. The later CD4^+^ TC rebound produced a biphasic pattern compatible with feedback from the expanding BC compartment. Temporal ordering alone does not prove causality, but it provides an in vivo context for the contact‐independent effect observed in co‐culture. Activated CD4^+^ TCs enhanced PBC differentiation in both direct and transwell cultures, indicating that cell contact was not required for this component of the response. Exogenous cholesterol reproduced the effect, whereas RORA inhibition in BCs and probucol pretreatment of TCs attenuated it, supporting a cholesterol‐sensitive, RORA‐associated route. The accompanying reduction in IL‐6 and IL‐23, minimal change in TNF‐α, and increase in IL‐10 under probucol‐treated conditions further suggest selective remodeling of the inflammatory milieu rather than indiscriminate cytokine suppression. One plausible mechanism is that cholesterol enhances BCR signaling through lipid‐raft assembly, thereby lowering the activation threshold [49]. Increased sterol demand may also engage SREBP2, which is required for effective humoral immunity [44], while diversion of the mevalonate pathway away from geranylgeranyl pyrophosphate could impair IL‐10‐producing Breg differentiation [64]. Together with enhanced FAO and defective IL‐2 signaling [45], these changes could shift BCs from homeostatic restraint toward pathogenic effector function. However, the cholesterol assay measured optical‐density signals rather than absolute flux, and no isotope‐resolved or transporter‐specific tracing was performed. Direct TC‐to‐BC cholesterol transfer therefore remains a hypothesis. Within this boundary, the data support a model in which soluble, cholesterol‐sensitive signals from primed CD4^+^ TCs promote BC differentiation, after which activated BCs may reinforce a second wave of CD4^+^ TC activation.

MCL1 appears to sustain the pathogenic TC state without acting as the principal driver of glycolysis. MCL1 inhibition reduced Ki67 and Th17‐associated proliferation readouts and shifted CD4^+^ TCs from *T*_em_ toward a naive phenotype, consistent with a role in cellular fitness and effector maintenance. By contrast, MCL1i did not broadly suppress HK2, LDHA, or GLUT1 and produced only modest changes in PFKFB3 and PKM2, unlike JAK1 or STAT3 inhibition. Although MCL1 is canonically regarded as an anti‐apoptotic protein, recent evidence has also revealed a distinct, apoptosis‐unrelated role for MCL1 in regulating mitochondrial metabolism. Specifically, in hepatocytes, MCL1 has been shown to directly bind acyl‐CoA synthetase long chain family member 1 (ACSL1), thereby facilitating the import and beta‐oxidation of long‐chain fatty acids into mitochondria, a process that would be expected to promote OXPHOS [50]. Whether this metabolic function is conserved in primary TCs, however, remains to be established. However, even if operative in TCs, any MCL1‐dependent facilitation of FAO would stand in direct contrast to the bioenergetic phenotype of pathogenic CD4^+^ *T*_em_ cells in ON, which was characterized by heightened glycolysis and diminished oxidative metabolism. This discordance implied that any metabolic role of MCL1 in facilitating FAO is unlikely to be the principal driver of its upregulation in this context. We therefore proposed that in autoimmune disorders such as ON and MS, the primary function of elevated MCL1 remained its canonical anti‐apoptotic activity, which was essential for sustaining the survival of pathogenic CD4^+^ *T*_em_ cells within the inflamed CNS. By contrast, in diseases such as RA or SLE, where TC pathology is driven by distinct metabolic programs that do not rely as heavily on MCL1‐mediated survival, the selective pressure for MCL1 elevation is absent, and its expression may remain unchanged or even decline. MCL1 is therefore best positioned here as a context‐dependent effector of TC persistence and phenotype maintenance within a broader JAK1‐STAT3‐associated program.

The therapeutic profile of UPA emerging from this study distinguishes it from conventional JAK inhibitors in several important respects. Whereas the efficacy of pan‐JAK inhibition in systemic autoimmune diseases is largely ascribed to the broad suppression of multiple cytokine pathways, our findings suggest that in ON and EAE, UPA operates through a more discrete mechanism. By targeting the JAK1‐pSTAT3‐MCL1 axis, UPA attenuates the pathogenic fitness of CD4^+^ *T*_em_ cells, an effect that coincides with diminished BC activation. This coordinated amelioration across both compartments suggests that UPA does not bluntly suppress inflammation but rather intercepts a pathogenic program that sustains reciprocal lymphocyte autoimmunity. Given that the JAK1‐MCL1 cascade is elevated in both ON and MS, early UPA intervention in ON may offer a unique opportunity to intercept disease evolution before widespread CNS autoimmunity becomes entrenched. Although our findings suggest that pharmacological JAK1 inhibition may attenuate neuroinflammation in ON, clinical translation of upadacitinib requires a cautious benefit‐risk assessment. Its efficacy is well established in systemic autoimmune diseases: 15 mg once daily improved clinical responses and inhibited radiographic progression in RA and was non‐inferior to adalimumab in psoriatic arthritis, whereas UC and CD regimens use 45 mg once daily for induction followed by 15 or 30 mg for maintenance [19, 51, 52, 53]. However, these doses cannot be extrapolated directly to ON. In an integrated analysis encompassing 27164 patient‐years, serious infection, herpes zoster, major adverse cardiovascular event, and venous thromboembolism rates were 1.3–4.6, 2.4–6.6, 0–0.5, and 0–0.9 per 100 patient‐years, respectively, with herpes zoster occurring more frequently than with adalimumab or methotrexate [54]. Upadacitinib‐mediated immunosuppression is expected to be exposure‐dependent rather than permanent because JAK1 blockade is reversible and the extended‐release formulation has a terminal half‐life of 9–14 h. Nevertheless, the interval required for infection risk to return fully to baseline after discontinuation remains undefined [55, 56]. Importantly, no topical, periocular, or intravitreal formulation, and no clinical study in ON, NMOSD, MOGAD, or MS, has been reported. The available ocular evidence is limited to small case series using oral upadacitinib for refractory uveitis [57]. Thus, local delivery remains speculative, and upadacitinib should currently be regarded as an experimental ON candidate rather than a demonstrated alternative to corticosteroids, conventional immunosuppressants, or disease‐specific biologics.

In summary, the contribution of cholesterol‐RORA crosstalk to BC fate decisions in neuroinflammation and the predictive value of MCL1 profiling in CD4^+^ *T*_em_ cells each warrant dedicated investigation. Prospective clinical evaluation of UPA in acute ON will be essential to validate the therapeutic relevance of these findings. Additionally, the potential of MCL1 profiling in CD4^+^ *T*_em_ cells to stratify patients or predict treatment response merits prospective evaluation.

## 4 Conclusion

In summary, our study delineates a pathogenic CD4^+^ *T*_em_ cell state in ON driven by a JAK1‐pSTAT3‐MCL1 signaling axis, which is functionally coupled to a cholesterol‐RORA‐mediated metabolic program in autoreactive BCs. Our findings suggest that this TC‐intrinsic metabolic rewiring contributes to instructing aberrant BC responses, thereby establishing a self‑amplifying T‑B inflammatory loop. Cross‑disease analyses implicate this axis as a conserved feature of allied autoimmune disorders. Notably, pharmacological intervention with UPA achieves dual correction of TC pathogenicity and BC dysfunction. These observations establish the JAK1‐pSTAT3‐MCL1 axis as a mechanistically grounded therapeutic target and provide preclinical evidence supporting UPA as a promising therapeutic strategy for ON and related neuroinflammatory conditions driven by coordinated T‑B dysregulation.

## 5 Methods

### 5.1 Human Donors

Healthy individuals and patients with optic neuritis were enrolled at Sun Yat‐sen University's Zhongshan Ophthalmic Center in Guangzhou, China, and blood samples were collected for single‐cell sequencing. The venous peripheral blood of patients with optic neuritis was collected at Shanghai Ninth People's Hospital, Shanghai Jiao Tong University School of Medicine, for flow cytometry analysis. All patients were in the active phase of the disease and provided informed consent. Patients with age and gender biases were excluded.

In accordance with the 2022 revised diagnostic criteria for optic neuritis, the diagnosis of optic neuritis was based on clinical manifestations, optical coherence tomography (OCT), magnetic resonance imaging (MRI), and visual evoked potentials [1]. The optic neuritis patients had acute or subacute disease at the initial stage and had not received any prior immunosuppressive or corticosteroid therapy before blood collection. The diagnosis was established through comprehensive clinical history, neurological evaluation, and characteristic findings on fundoscopy, OCT, and orbital MRI. Disease activity was assessed based on the presence of acute optic nerve dysfunction, including monocular or binocular visual acuity decline of acute or subacute onset (typically within 7 days to 3 weeks), relative afferent pupillary defect (RAPD), acquired dyschromatopsia, and fundoscopic signs such as optic disc edema (in anterior optic neuritis) or normal‐appearing disc (in retrobulbar optic neuritis). Additional paraclinical criteria included inter‐eye difference in peripapillary retinal nerve fiber layer (pRNFL) >5% or >5 µm, or in macular ganglion cell inner plexiform layer (mGCIPL) >4% or >4 µm on OCT, and contrast enhancement of the symptomatic optic nerve on fat‐suppressed T1‐weighted MRI. Patients were included if they experienced their first episode of optic neuritis without evidence of alternative diagnoses (e.g., ischemic optic neuropathy, compressive lesions, or hereditary optic neuropathies). Exclusion criteria were prior use of corticosteroids or other immunomodulatory therapies, history of multiple sclerosis or neuromyelitis optica spectrum disorder before the index event, and any systemic infection or malignancy that could mimic optic neuritis. Peripheral blood samples were collected at the time of acute presentation (within 7 days of symptom onset) before initiation of high‐dose methylprednisolone therapy. The Zhongshan Ophthalmic Center's Medical Ethics Committee in Guangzhou examined and approved each procedure (ID: 2023KYPJ095‐2).

### 5.2 Mice

C57BL/6J mice (6–8 weeks old, average weight 20 g) were obtained from the Animal Experiment Center (Shanghai, China). All mice were kept under specific pathogen‐free conditions in accordance with the guidelines approved by the Animal Care and Use Committee of Shanghai Jiao Tong University (SH9H‐2026‐A1360‐1).

### 5.3 Establishment of the EAE Mice Model

After anesthesia, each C57BL/6J mouse received a subcutaneous injection of 200 µL of a mixture containing 2 mg/mL myelin oligodendrocyte glycoprotein (MOG_35‐55_, GL Biochem, Shanghai, China) peptide and 2.5 mg/mL of Mycobacterium tuberculosis strain H37Ra (Difco, San Jose, CA, USA) in a 1:1 volume ratio. On the immunization day and again two days later, 0.25 µg of pertussis toxin (PTX; List Biological Laboratories, Campbell, CA, USA) in PBS was intraperitoneally injected.

A daily clinical score (0–5) was assigned to assess EAE severity based on the following criteria: 0, healthy; 0.5, tail weakness; 1, partial tail paralysis; 1.5, full tail paralysis; 2, flaccid tail; 2.5, flaccid tail and hind limb weakness; 3, partial hind limb paralysis; 4, total hind limb paralysis; 5, complete forelimb and hindlimb paralysis [58]. Mouse spinal cords were fixed in paraformaldehyde for 48 h, paraffin‐embedded, sectioned (4 µm), stained with H&E and LFB, sealed, and photographed. Microscopic images were assessed pathologically as described [59]. Blinded histological assessment was carried out.

### 5.4 Treatment and Isolation of Cells From EAE Mice

Following vaccination, mice were given daily intraperitoneal injections of either vehicle control (PBS, *n* = 6) or upadacitinib (10 mg/kg/day, *n* = 6) through day 28 after immunization. Compound upadacitinib was purchased from MedChemExpress (Cat.No. HY‐19569). Digested cells from mouse lymph nodes (LN), spleens (SP), and spinal cord were collected and filtered through a 70‐µm filter. In vitro, cells were isolated and cultured for 72 h with either no treatment (blank), MOG_35‐55_ (20 µg/mL) alone, MOG_35‐55_ plus upadacitinib (5 µm), or MOG_35‐55_ plus A‐1210477 (10 µm).

### 5.5 Adoptive Transfer Experiment

On day 11 after EAE induction, draining lymph nodes were collected from donor mice and processed into single‐cell suspensions. Cells were cultured at 37°C for 72 h with MOG_35‐55_ (20 µg/mL), recombinant mouse IL‐6 (20 ng/mL), IL‐12 (10 ng/mL), and IL‐23 (10 ng/mL), with or without UPA (5 µm). On day 14, viable CD4^+^ TCs were purified from the cultured cells by flow cytometric sorting. Cells from six donor mice were pooled for each treatment group, and each naïve C57BL/6J recipient mouse received 1 × 10^7^ viable CD4^+^ TCs by intravenous tail‐vein injection. Recipient mice were subsequently monitored for body weight and clinical signs of EAE, and spinal cords were collected on day 42 for histological and flow‐cytometric analyses.

### 5.6 Plasmid Construction and Electroporation

Full‐length murine Mcl1 cDNA was cloned into the pLV3‐CMV‐3×FLAG‐Puro vector to generate pLV3‐CMV‐Mcl1‐3×FLAG‐Puro. Primary CD4^+^ TCs isolated from wild‐type mice were electroporated at 1 × 10^6^ cells per reaction with 4 µg of pLV3‐CMV‐Mcl1‐3×FLAG‐Puro plasmid using the Neon Transfection System (Invitrogen). Immediately after electroporation, cells were transferred to prewarmed complete medium in 24‐well plates. Cells electroporated with the corresponding empty vector served as controls. MCL1 overexpression was confirmed by flow cytometry after electroporation.

### 5.7 siRNA‐Mediated JAK1 Knockdown

Three siRNA duplexes targeting murine Jak1 were chemically synthesized and HPLC‐purified by Shanghai Yigou Biotechnology Co., Ltd. (Shanghai, China). The sense/antisense sequences were as follows: Jak1‐mouse‐1668, 5′‐GCUUUGAUCGGAUCCUUAATT‐3′/5′‐UUAAGGAUCCGAUCAAAGCTT‐3′; Jak1‐mouse‐485, 5′‐GAGCAAGACGGACAUGAUATT‐3′/5′‐UAUCAUGUCCGUCUUGCUCTT‐3′; and Jak1‐mouse‐214, 5′‐GAAGGAGAUAGAGAUCUUATT‐3′/5′‐UAAGAUCUCUAUCUCCUUCTT‐3′. A non‐targeting siRNA duplex (sense, 5′‐UUCUCCGAACGUGUCACGUTT‐3′; antisense, 5′‐ACGUGACACGUUCGGAGAATT‐3′) was used as the negative control.

Primary murine CD4^+^ TCs were transfected separately with each candidate siRNA using siRNA SuperTrans reagent according to the manufacturer's instructions. For each well of a 24‐well plate, 40 pmol siRNA and 2 µL transfection reagent were separately diluted in 50 µL serum‐free Opti‐MEM and incubated for 5 min at room temperature. The two solutions were combined, incubated for 20 min, and added to cells cultured in 500 µL medium. The medium was replaced after 6 h. Knockdown efficiency was evaluated by qRT‐PCR after transfection using the following Jak1 primers: forward, 5′‐TATTGGGTGGCCAGAAGCAG‐3′; reverse, 5′‐GTGGTTCATGAGGTCTCGCA‐3′. The siRNA producing the greatest reduction in Jak1 expression was designated siJAK1 and used in subsequent experiments. For functional assays, transfected cells were stimulated with MOG_35‐55_ (20 µg/mL) and analyzed 72 h after transfection.

### 5.8 Co‐Culture and Perturbation Assays

Single‐cell suspensions were prepared from the lymph nodes of EAE mice 11 days after immunization. CD4^+^ TCs and CD19^+^ BCs were purified using Mouse CD4^+^ T Cell Isolation Kit (STEMCELL) and Mouse CD19 Positive Selection Kit II (STEMCELL). Cell purity was confirmed by flow cytometry and was required to exceed 90%–95%. Cells were cultured for 7 days in complete RPMI 1640 medium containing 10% fetal bovine serum, penicillin‐streptomycin, *L*‐glutamine, and β‐mercaptoethanol at 37°C in a humidified atmosphere containing 5% CO_2_.

Purified CD19^+^ BCs were seeded at 1 × 10^6^ cells per well. For direct co‐culture, CD4^+^ TCs were added to the B cell cultures at a TC: BC ratio of 1:1. For contact‐independent co‐culture, CD19^+^ BCs were placed in the lower chamber, and an equivalent number of CD4^+^ TCs was placed in the upper chamber of a polycarbonate transwell insert with a pore size of 0.4 µm. The culture medium, cell numbers, and final culture volumes were kept identical between the direct and transwell conditions.

To investigate the contribution of cholesterol‐sensitive signaling and RORA activity, BCs were treated with SR3335 as the RORA inverse agonist (RORAi; 5 µm; TargetMol, USA, T5160) for cholesterol supplementation or co‐culture. Cholesterol (Water Soluble; TargetMol, USA, T40801) was added at a final concentration of 200 mg/L. For probucol experiments, CD4^+^ TCs were pretreated with probucol at 10 µm for 24 h, washed 3 times with complete medium, and subsequently introduced into either direct or transwell co‐culture. Equivalent vehicle concentrations were included in all control groups.

### 5.9 Flow Cytometry

Harvested cells were first stained with live/dead cell dye (BioLegend) for 10 min at 4°C. Subsequently, the cells were treated with ionomycin (10 µg/mL), brefeldin A (BFA, 20 µg/mL), and phorbol 12‐myristate 13‐acetate (PMA, 1 µg/mL) for 5 h at 37°C. After stimulation, surface antibodies against CD3, CD4, CD8, CD45, CD11b, CD19, CD138, B220, and CD25 (BioLegend) were added, and the mixture was incubated for 15 min at 4°C. Following membrane permeabilization and fixation, intracellular antibody labeling was performed using antibodies against IFN‑γ, IL‑17A, FOXP3, pSTAT3, MCL1, IL‑10 (BioLegend), HK2, PFKFB3, LDHA, GLUT1, and PKM2 (Abcam) overnight at 4°C. The stained cells were then analyzed by flow cytometry, and the data were interpreted using FlowJo software (version 10.8.1, BD Biosciences).

### 5.10 CFSE Proliferation Assay

CD4^+^ TCs were sorted by flow cytometry, labeled with 2.5 µm CFSE (BD Biosciences) for 10 min at 37°C under continuous mixing, and quenched with chilled complete growth medium. The labeled cells were subsequently stimulated with CD3/28 Dynabeads (GIBCO) either alone or in the presence of 1–10 µm Upadacitinib, or 1–20 µm A‐1210477 (MedChemExpress, Cat.No. HY‐12468), or 0.5–5 µm Stattic (TargetMol, USA, T6308), or 1–20 µm Probucol (TargetMol, USA, T0254). After 72 h of incubation, CD4^+^ TC proliferation was assessed by flow cytometry. Sorted BCs were similarly labeled with 2.5 µm CFSE and stimulated with CpG ODN1826 (TargetMol, USA, T74509), CD40L, IL‐4, and IL‐21, with or without 1–10 µm Upadacitinib or 1–10 µm SR3335. Following a 4‐day incubation, BC proliferation was evaluated by flow cytometry.

### 5.11 Apoptosis Assay

After treatment, cells were collected by centrifugation at 1500 rpm for 5 min, washed twice with pre‐cooled PBS, and resuspended in 1× Binding Buffer at 1–5 × 10^6^ cells/mL. Aliquots containing 1–5 × 10^5^ cells in 100 µL were incubated with 5 µL Annexin V‐FITC and 5 µL 7‐AAD (Goonie Biotechnology, Guangzhou, China) for 10–15 min at room temperature in the dark. Subsequently, 400 µL of 1× Binding Buffer was added, and samples were analyzed by flow cytometry within 1 h.

### 5.12 PBMCs Isolation for scRNA‐Seq

Peripheral venous blood was drawn from healthy donors and individuals diagnosed with optic neuritis. Isolation of peripheral blood mononuclear cells (PBMCs) was performed using Ficoll–Hypaque density gradient centrifugation (GE Healthcare, Chicago, IL, USA) in accordance with standard protocols. A cell viability exceeding 85% was observed for all samples.

### 5.13 Single‐Cell Data Analysis From the Public Database

Publicly available single‐cell RNA‐seq data of multiple sclerosis (MS), giant cell arteritis (GCA), Kawasaki disease (KD), psoriasis (Pso), primary Sjögren's syndrome (pSS), rheumatoid arthritis (RA), systemic lupus erythematosus (SLE), ulcerative colitis (UC), Crohn's disease, Vogt–Koyanagi–Harada (VKH) disease and Behçet's disease (BD) were collected. For MS, PBMC datasets GSE138266, GSE233917, GSE239626 and paired healthy control PBMC datasets GSE220189, GSE213516, as well as cerebrospinal fluid (CSF) datasets GSE138266, GSE163005 and paired healthy control CSF datasets GSE134578, GSE202410, were obtained from the Gene Expression Omnibus. GCA data were from GSE198891, KD data from GSE200743, Pso data from GSE182244, pSS data from GSE157278, SLE data from GSE137029, and UC and Crohn's disease data from GSE125527, and RA data were from HRA000155 (GSA for Human). The raw data from the repositories were used as input and processed using the Scanpy package.

### 5.14 scRNA‐Seq Data Processing

Single‐cell RNA sequencing (scRNA‐seq) libraries were prepared using the Chromium Single Cell 5′ Library and Gel Bead Kit (10× Genomics, 120237) following the manufacturer's instructions with minor modifications. Specifically, after washing with 0.04% BSA buffer (0.02 g BSA dissolved in 50 mL deionized PBS), cells were retained within the droplets. Reverse transcription, emulsion fragmentation, PCR amplification, and barcoded cDNA purification with Dynabeads were then performed sequentially. The amplified cDNA was used to construct a 5′ gene expression library. For 150 bp paired‐end sequencing, 50 ng of the amplified cDNA was fragmented, end‐repaired, size‐selected twice with SPRiSelect beads, and sequenced on the NovaSeq platform (Illumina NovaSeq6000). Raw sequencing data were processed with CellRanger software v7.0.1 (10× Genomics). Data integration and clustering were performed using Python (v3.10) and the Scanpy package (v1.11.1). Quality control filtering retained cells expressing 300 to 4000 genes with less than 15% mitochondrial reads. Cells from human samples expressing high levels of HBB, HBA1, as well as various light and heavy chain transcripts, indicative of red blood cell contamination, were removed. Similarly, cells expressing HHB‐A1 and HHB‐BS, recognized as erythrocyte markers, were excluded. Batch effects across samples were corrected using the Harmonypy package (v0.0.10) [60].

### 5.15 Dimensionality Reduction and Cluster Analysis

For scRNA‑seq analysis, raw data were processed with the ‘cellranger count’ function of CellRanger (10× Genomics, Pleasanton, CA, USA) and mapped to the human reference genome. Aggregation of multiple samples was performed using ‘cellranger aggr’ to generate a quantitative expression matrix. Quality control criteria included a mitochondrial transcript percentage below 15% and a per‑cell gene count between 200 and 4000. Log‑normalization was applied using the ‘scanpy.pp.normalize_total’ function, and batch effects were corrected with the Harmonypy algorithm. Major cell clusters were identified by the ‘scanpy.tl.leiden’ function (resolution = 0.5), and data visualization was carried out using the Uniform Manifold Approximation and Projection (UMAP) algorithm via the ‘scanpy.pl.umap’ function. Differentially expressed genes (DEGs) for each cell type were determined using the ‘scanpy.tl.rank_genes_groups’ function combined with the Wilcoxon rank‑sum test and Bonferroni correction, applying thresholds of |log_2_ fold change| > 0.25 and adjusted *p* < 0.05.

### 5.16 GO Analysis

Gene Ontology (GO) analysis was carried out via Metascape (www.metascape.org), and *p*‐values were determined by the cumulative hypergeometric test. Across different cell types, the 50 most significantly enriched terms were plotted with pheatmap (v1.0.12) and ggplot2 (v3.2.1), from which 5–15 relevant pathways were selected among the top 100 [61].

### 5.17 Cell–Cell Communication

Cell–cell communication analysis was performed using normalized expression matrices generated from Seurat, with further processing and visualization conducted via the R package CellChat (v1.5.0) [62]. Ligand–receptor pair annotations were obtained from CellChatDB, a literature‐curated database that provides known interactions between ligands and receptors for both mouse and human [63]. For each pair of PBMC cell types, we calculated the total number of ligand–receptor interactions based on CellPhoneDB annotations and visualized the interaction count matrix as a heatmap using the ggplot2 package.

### 5.18 Gene Expression Correlation Analysis

Linear relationships between genes were quantified by computing Spearman's R (R) on the normalized single‐cell matrix. A *p*‐value below 0.05 was considered evidence of significant co‐expression among the assayed cells.

### 5.19 ELISA

Serum levels of IgG were quantified with respective mouse ELISA kits (Thermo Fisher Scientific, Waltham, MA, USA) following the manufacturer's instructions. In addition, the concentrations of TNF‐α, IL‐10, IL‐23, and IL‐6 in cell culture supernatants were measured using corresponding mouse ELISA kits (Jiang Lai Biological, Shanghai, China), according to the manufacturer's protocols.

### 5.20 Statistics

All experiments were repeated at least three times. Data were processed and visualized using GraphPad Prism (version 8.0.2, GraphPad Software, La Jolla, CA, USA). Results are presented as mean ± standard deviation (SD). Statistical comparisons were made using one‐way or two‐way ANOVA, unpaired two‐tailed Student's *t‐*test or unpaired two‐tailed Wilcoxon rank‐sum test, as appropriate. A *p*‐value < 0.05 was considered statistically significant, with significance levels denoted as *p < 0.05, **p < 0.01, ***p < 0.001, ****p < 0.0001, and ns for not significant.

## Author Contributions

G.J., Z.L., Q.J., L.H., Y.P., H. Y., W.Z. and W.S. designed research; G.J., Q.J., L.H., Y.P., X.L., T.Y., H.H, J.L. and Z.L. performed research; G.J., Q.J., L.H., Y.P., X.L., H.H., and T.Y. analyzed data; and G.J., Q.J., Z.L. and W.S. wrote the paper. J.L., Y.G., and W.Z. provided intellectual input into the experiments throughout the study, provided comments, and helped edit the manuscript. All authors have read and approved the final manuscript.

## Ethics Statement

All animal experiments were approved by the Laboratory Animal Ethics Committee of Shanghai Ninth People's Hospital, Shanghai Jiao Tong University School of Medicine (Approval No.: SH9H‐2026‐A1360‐1). All procedures were performed in accordance with the National Institutes of Health Guide for the Care and Use of Laboratory Animals and the Chinese regulations on the administration of laboratory animals. Every effort was made to minimize animal suffering and to reduce the number of animals used.

## Conflicts of Interest

The authors declare no conflicts of interest.

## Supporting information




**Supporting File 1**: advs77701‐sup‐0001‐SuppMat.docx.

**Supporting File 2**: advs77701‐sup‐0002‐TableS1.xlsx.

## Data Availability

Processed single‐cell RNA‐seq data have been deposited at the National Genomics Data Center (NGDC) and are publicly available. Accession number: OMIX016517.
