# Supplementary material for: Upadacitinib Restrains the Pathogenic Fitness of CD4+ T Cells and Aberrant B Cell Programming in Optic Neuritis
Source: Adv Sci (Weinh). 2026 Sep 9:e77701. Online ahead of print. doi: 10.1002/advs.77701 (PMC13558900; doi:10.1002/advs.77701)
Supplement: Supplementary file 1 [file ADVS-9999-e77701-s001.docx]

# **Supplementary Information**

**Upadacitinib Restrains the Pathogenic Fitness of CD4^+^ T Cells and Aberrant B Cell Programming in Optic Neuritis**

*Gengchen Jiang, Qi Jiang, Lian Han, Yue Peng, Xingyu Liu, Tong Yang, Hao Huang, Jiaying Liu, Yuan Gao, Hui Yang, Wenjun Zou*, Zhaohuai Li*, Wenru Su**

G. Jiang, Q. Jiang, X. Liu, H. Huang, Y. Gao, Z. Li, W. Su

Department of Ophthalmology, Ninth People’s Hospital, Shanghai Jiao Tong University School of Medicine, Shanghai 200011, China.

E-mail: [lizhh55@mail2.sysu.edu.cn;](mailto:lizhh55@mail2.sysu.edu.cn,) [suwenru@sjtu.edu.cn](mailto:suwenru@sjtu.edu.cn).

G. Jiang, Q. Jiang, X. Liu, H. Huang, Y. Gao, Z. Li, W. Su

State Key Laboratory of Eye Health, Department of Ophthalmology, Shanghai Ninth People’s Hospital, Shanghai Jiao Tong University School of Medicine, Shanghai 200001, China.

L. Han, T. Yang, H. Huang

The First Affiliated Hospital of Bengbu Medical University, Bengbu Medical University, Bengbu 233030, China.

Y. Peng

School of Clinical Medicine, Qinghai University, Xining 810008, China.

H. Huang

Department of Ophthalmology, Zhuzhou Hospital Affiliated to Xiangya School of Medicine, Central South University, Zhuzhou 412000, China.

J. Liu, *H. Yang,* Z. Li

State Key Laboratory of Ophthalmology, Zhongshan Ophthalmic Center, Sun Yat-sen University, Guangdong Provincial Key Laboratory of Ophthalmology and Visual Science, Guangdong Provincial Clinical Research Center for Ocular Diseases, Guangzhou 510060, China.

W. Zou

Department of Ophthalmology, Wuxi No.2 People’s Hospital, Jiangnan University Medical Center, Wuxi 214002, China.

Gengchen Jiang, Qi Jiang, Lian Han, Yue Peng and Xingyu Liu contributed equally to this work.

**Supplementary Figures**

**Figure S1**

**Figure S1. Significant perturbations in immune cell subsets associated with ON pathogenesis:**

(A) UMAP plots of immune cell populations of major PBMC groups split by individual samples. HCF, female healthy controls; HCM, male healthy controls; ON, optic neuritis.

(B) UMAP plots of immune cell populations of major PBMC groups split by groups. BC, B cell; PBC, plasma cell; TC, T cell; NK, natural killer cell; Mono, monocyte; DC, dendritic cell; Mgk, megakaryocyte.

(C) Stacked bar chart displaying the proportional distribution of major cell populations in PBMCs among HC, HCM, HCF, ON.

(D) ROGUE analysis of cellular heterogeneity across PBMC subpopulations.

(E) Volcano plot of ON-related differentially expressed genes (ON-DEGs) derived from all major cells.

(F) GO analysis of upregulated ON-DEGs of all major cells.

(G) GO analysis of downregulated ON-DEGs of all major cells.

(H) GO analysis of upregulated ON-DEGs of T cells.

(I) Bubble plot showing representative GO terms enriched in the major immune cell subsets.

**Figure S2**

**Figure S2. Transcriptomic landscape and functional dynamics of T cell subpopulations in ON, with a particular focus on CD4^+^ Tem cell differentiation:**

(A) Stacked bar chart displaying the proportional distribution of T cell subsets in PBMCs among HC, HCM, HCF, ON.

(B) Pseudotime trajectory analysis of CD8^+^ T cells, colored by celltype. NCD8, naive CD8^+^ T; CD8^+^ effector memory, CD8^+^ Tem cells; CTL, CD8^+^ cytotoxic T cells.

(C) Pseudotime trajectory analysis of CD8^+^ T cells, colored by pseudotime.

(D) Histogram showing the cell population distribution corresponding to panels (B) and (C).

(E) Bubble plot showing representative GO terms enriched in downregulated ON-DEGs of the T cell subsets.

(F) Volcano plot of ON-DEGs derived from all CD4^+^ Tem cells.

(G) GO analysis of the overlapping genes between genes downregulated or stably expressed in NCD4 cells and upregulated ON-DEGs in CD4^+^ Tem cells.

(H) Venn diagram illustrating the overlap between upregulated ON-DEGs in NCD4 cells and downregulated ON-DEGs in CD4^+^ Tem cells.

(I) GO analysis of overlapping genes derived from (H).

(J) Palantir pseudotime computed with NCD4 cells as root and component 2 as the ordering axis.

**Figure S3**

**Figure S3. Single-cell atlas of peripheral blood mononuclear cells from multiple sclerosis patients and healthy controls:**

(A) UMAP projection showing the clustering of major cell subsets across healthy control (HC) and multiple sclerosis (MS) samples.

(B) Bar plots quantifying the relative proportions of each major cell subset in the HC and MS groups. BC, B cell; PBC, plasma cell; TC, T cell; NK, natural killer cell; Mono, monocyte; DC, dendritic cell; Mgk, megakaryocyte.

(C) UMAP projections depicting the expression of canonical marker genes for major cell subsets in HC and MS human samples. NCD4, naïve CD4^+^ T; CD4^+^ Tcm, CD4^+^ central memory T; CD4^+^ Tem; CD4^+^ effector memory T; Treg, CD4^+^ regulatory T; NCD8, naïve CD8^+^ T; CD8^+^ Tem, CD8^+^ effector memory; CTL, CD8^+^ cytotoxic T cells; DNT, CD4^-^CD8^-^ T; Pro-T, proliferative T.

(D) UMAP plot illustrating the clustering of all T cell subsets identified from HC and MS patients. (E) Bar plots showing the relative proportions of each major T cell subset in HC versus MS patients.

(F) UMAP projections visualizing the expression of canonical markers for T cell subsets in HC and MS human samples.

**Figure S4**

**Figure S4. Single-cell atlas of cerebrospinal fluid from multiple sclerosis patients and healthy controls:**

(A) UMAP projection showing the clustering of major cell subsets across healthy controls (HC) and multiple sclerosis (MS) samples.

(B) Bar plots quantifying the relative proportions of each major cell subset in the HC and MS groups. BC, B cell; PBC, plasma cell; Memory CD4, memory CD4^+^ T cell; Memory CD8, memory CD8^+^ T cell; NK, natural killer cell; Mono, monocyte; cDC1, type 1 conventional dendritic cell; cDC2, type 2 conventional dendritic cell; pDC, plasmacytoid dendritic cell; CD14^+^ Mono, CD14^+^ monocyte; CD16^+^ Mono, CD16^+^ monocyte.

(C) UMAP projections showing the expression of canonical marker genes for major cell subsets in HC and MS samples.

**Figure S5**

**Figure S5. Single-cell atlas of peripheral blood mononuclear cells from patients with distinct autoimmune diseases and healthy controls:**

(A) UMAP projection showing the clustering of T cell subsets across healthy controls (HC) and patients with various autoimmune diseases, including giant cell arteritis (GCA), Kawasaki disease (KD), primary Sjögren's syndrome (pSS), rheumatoid arthritis (RA), psoriasis (Pso), Crohn’s disease (CD), ulcerative colitis (UC), Behçet’s disease (BD), systemic lupus erythematosus (SLE), and Vogt-Koyanagi-Harada disease (VKH).

(B) UMAP projections showing the expression of canonical marker genes for T cell subsets in HC and autoimmune disease patients.

(C-D) Violin plots of JAK1 and MCL1 expression levels in CD4^+^ T cell subsets based on scRNA-seq data of peripheral blood mononuclear cells (PBMCs) from HC (n = 69) and GCA patients (n = 12).

(E-F) Violin plots of JAK1 and MCL1 expression levels in CD4^+^ T cell subsets based on PBMC scRNA-seq data from HC (n = 69) and KD patients (n = 3).

(G-H) Violin plots of JAK1 and MCL1 expression levels in CD4^+^ T cell subsets based on PBMC scRNA-seq data from HC (n = 69) and pSS patients (n = 3).

(I-J) Violin plots of JAK1 and MCL1 expression levels in CD4^+^ T cell subsets based on PBMC scRNA-seq data from HC (n = 69) and RA patients (n = 21).

(K-L) Violin plots of JAK1 and MCL1 expression levels in CD4^+^ T cell subsets based on PBMC scRNA-seq data from HC (n = 69) and Pso patients (n = 3).

(M-N) Violin plots of JAK1 and MCL1 expression levels in CD4^+^ T cell subsets based on PBMC scRNA-seq data from HC (n = 69) and CD patients (n = 8).

(O-P) Violin plots of JAK1 and MCL1 expression levels in CD4^+^ T cell subsets based on PBMC scRNA-seq data from HC (n = 69) and UC patients (n = 7).

(Q-R) Violin plots of JAK1 and MCL1 expression levels in CD4^+^ T cell subsets based on PBMC scRNA-seq data from HC (n = 69) and SLE patients (n = 51).

(S-T) Violin plots of JAK1 and MCL1 expression levels in CD4^+^ T cell subsets based on PBMC scRNA-seq data from HC (n = 69) and BD patients (n = 13).

(U-V) Violin plots of JAK1 and MCL1 expression levels in CD4^+^ T cell subsets based on PBMC scRNA-seq data from HC (n = 69) and VKH patients (n = 35).Significance was evaluated using unpaired two-tailed Wilcoxon rank-sum test (Mann-Whitney U test). *P<0.05, **P<0.01, ***P<0.001, ****P<0.0001, and ns for not significant.

**Figure S6**

**Figure S6. Aberrant crosstalk between CD4^+^ Tem and B cells and metabolic reprogramming in ON:**

(A-D) Reconstructed cell communication landscape of immune cells in HC (left) and ON patients (right).

(E-H) COMPASS analysis identifies distinct metabolic shifts in CD4^+^ Tem cells associated with optic neuritis (ON).

(I) MEBOCOST chord diagram showing metabolite transfer among major immune cells and T cell subsets in healthy controls (HC).

(J) Violin plots displaying the expression levels of SLC2A3, S1PR1, and PTGER4 in major immune cells and T cell subsets from ON patients and healthy controls (HC).

(K) GO pathway enrichment network plot based on GSEA of all genes in B cells.

(L) Volcano plots showing differentially expressed genes (DEGs) in distinct B cell subtypes of ON patients, with a focus on the JAK1 gene.

**Figure S7**

**Figure S7. Flow cytometry gating strategy for identifying immune cell subsets:**

(A) Flow cytometry gating strategy for identifying specific T cell subsets.
(B) Flow cytometry gating strategy for identifying microglia subsets.
(C) Flow cytometry gating strategy for identifying specific B cell subsets.

**Figure S8**

**Figure S8. Comparative flow cytometry analysis of B cell, plasma cell, and CD4^+^ T cell subsets in EAE and UPA mice:**

(A-D) Proportions of B220^−^CD138^+^ plasma cells, CD19^+^CD86^+^ B cells, CD19^+^ MHCII^+^ B cells, and CD69^+^CD19^+^ B cells in the spinal cords of EAE mice (EAE) and upadacitinib-treated EAE mice (UPA) (n = 6 per group).

(E-H) Comparison of IFN-γ, IL-17A, and FOXP3 expression in CD4^+^ T cells and the proportion of B220^−^CD138^+^ plasma cells in the spleens of EAE and UPA-treated EAE mice (n = 6 per group).

**Figure S9**

**Figure S9. UPA modulates T and B cell immune responses in vitro and attenuates adoptive transfer EAE in vivo:**

(A-E) In vitro comparison of IFN-γ, IL-17A, and FOXP3 expression in CD4^+^ T cells, as well as the frequencies of CD19^+^IL-10^+^ regulatory B cells (Breg) and B220^−^CD138^+^ plasma cells among normal control (NC), MOG35-55 (MOG), and MOG+upadacitinib (UPA) groups (n = 6 per group).

(F) CFSE dilution assay showing the effects of different UPA concentrations on the proliferation of mouse CD19^+^ B cells (n = 3 per group).

(G) Flow cytometric analysis of cell apoptosis. Cells were divided into the non-stimulated control group, MOG_35-55_ single-treatment group, and MOG_35-55_ combined with gradient concentrations of UPA group. Cell apoptosis was assessed via Annexin V and 7-AAD staining and subsequent flow cytometry detection (n = 3 per group).

(H) Daily clinical scores of EAE mice receiving adoptive transfer of MOG_35-55_-primed T cells with or without UPA treatment (n = 6 per group).

(I) Daily relative body weight changes of EAE mice receiving adoptive transfer of MOG_35-55_-primed T cells with or without UPA treatment (n = 6 per group).

(J-K) Proportions of CD4^+^IFN-γ^+^ Th1 cells and CD4^+^IL-17A^+^ Th17 cells in the spinal cords of mice receiving adoptive transfer of MOG35-55-stimulated T cells with or without UPA pretreatment (n = 6 per group).

(L) CFSE dilution assay showing the effects of different MCL1i concentrations on the proliferation of mouse CD4^+^ T cells (n = 3 per group).

Data are shown as mean ± SD. Significance was evaluated using two-way ANOVA. *P<0.05, **P<0.01, ***P<0.001, ****P<0.0001, and ns for not significant.

**Figure S10**

**Figure S10. In vitro characterization of CD4^+^ T cell functional and phenotypic changes modulated by the JAK1-MCL1 pathway under MOG_35-55_ stimulation:**

(A-C) In vitro comparison of IFN-γ, IL-17A, and FOXP3 expression in CD4^+^ T cells among normal control (NC), MOG_35-55_ (MOG), and MOG_35-55_+MCL1 inhibitor (MCL1i) groups (n = 6 per group).

(D) Flow cytometric analysis of CD4^+^MCL1^+^ T cells under indicated in vitro culture conditions: NC, MOG_35-55_+vector (MOG), MOG_35-55_+MCL1 overexpression (OE-MCL1), and MOG_35-55_+OE-MCL1+MCL1i (MCL1i) (n = 6 per group). MOG upregulated MCL1 expression, which was further enhanced by OE-MCL1 and abrogated by MCL1i.

(E-G) Representative flow cytometry plots showing the percentages of CD4^+^IL-17A^+^ T cells (Th17), CD4^+^IFN-γ^+^ T cells (Th1) and CD4^+^FOXP3^+^ T cells (Treg) in the NC, MOG, MOG+OE-MCL1, MOG+upadacitinib (UPA), and MOG+OE-MCL1+UPA groups (n = 6 per group).

(H) Flow cytometric analysis of apoptosis. Cells were left untreated (Non-stimulated control), treated with MOG, or co-treated with MOG_35-55_ plus gradient concentrations of MCL1i. After staining with Annexin V and 7-AAD, apoptotic cell proportions were analyzed by flow cytometry (n = 3 per group).

(I) Representative flow cytometry plots showing the percentages of CD4^+^Ki67^+^ proliferating T cells in the NC, MOG, and MOG_35-55_-stimulated groups co-treated with gradient concentrations of MCL1i (n = 6 per group).

(J) Representative contour plots showing the proportions of CD4^+^ naive T cells (NCD4, CD4^+^CD44^-^CD62L^+^), CD4^+^ effector memory T cells (CD4^+^Tem, CD4^+^CD44^+^CD62L^-^), and CD4^+^ central memory T cells (CD4^+^Tcm, CD4^+^CD44^+^CD62L^+^) in the NC, MOG, and MCL1i group (n = 6 per group).

**Figure S11**

**Figure S11. JAK1-STAT3 signaling contributes to the regulation of glycolytic key molecules in CD4^+^ T cells under MOG_35-55_ stimulation in vitro:**

(A-D) Flow cytometric analysis of glycolysis-related molecules (HK2, PFKFB3, LDHA, GLUT1, and PKM2) in CD4^+^ T cells under in vitro culture conditions. Cells were divided into different treatment groups as follows: (A) non-stimulated control (NC), MOG_35-55_ (MOG), and MOG+siJAK1 groups; (B) NC, MOG, and MOG+Stattic groups; (C) NC, MOG, and MOG+upadacitinib (UPA) groups; (D) NC, MOG, and MOG+MCL1 inhibitor (MCL1i) groups. Representative flow cytometry plots show the percentages of CD4^+^HK2^+^, CD4^+^PFKFB3^+^, CD4^+^LDHA^+^, CD4^+^GLUT1^+^, and CD4^+^PKM2^+^ T cells in each group (n = 6 per group).

**Figure S12**

**Figure S12. Effects of mechanism inhibitors and upadacitinib on T and B cell proliferation and functional subsets in vitro:**

(A) CFSE dilution analysis showing the effect of various concentrations of stattic on the proliferation of mouse CD4^+^ T cells (n = 3 per group).

(B) CFSE dilution analysis showing the effect of various concentrations of RORA inhibitor (RORAi) on the proliferation of mouse CD19^+^ B cells (n = 3 per group).

(C) CFSE dilution analysis showing the effect of various concentrations of probucol on the proliferation of mouse CD4^+^ T cells (n = 3 per group).

(D) CFSE dilution analysis showing the effect of various concentrations of upadacitinib (UPA) on the proliferation of human CD4^+^ T cells (n = 3 per group).

(E) CFSE dilution analysis showing the effect of various concentrations of UPA on the proliferation of human CD19^+^ B cells (n = 3 per group).

(F) CFSE dilution analysis showing the effect of various concentrations of MCL1 inhibitor (MCL1i) on the proliferation of human CD4^+^ T cells (n = 3 per group).

(G-L) Proportions of CD3^+^CD8^-^IL-17A^+^ T cells (Th17), CD3^+^CD8^-^IFN-γ^+^ T cells (Th1), CD3^+^CD8^-^CD25^+^FOXP3^+^ T cells (Treg), CD20^-^CD38^+^ plasma cells, CD3^+^CD8^-^pSTAT3^+^ T cells and CD3^+^CD8^-^MCL1^+^ T cells. Peripheral blood mononuclear cells (PBMCs) from patients with optic neuritis (ON) were cultured in vitro and divided into untreated (NC), human anti-CD3/CD28 stimulation, and human anti-CD3/CD28 plus UPA treatment (UPA) groups (n = 6 per group).

**Figure S13**

**Figure S13. Effects of upadacitinib and MCL1i on human immune cell profiles and related metabolic activities in vitro:**

(A) Proportions of CD19^+^IL-10^+^ regulatory B cells (Breg) in in vitro cultured peripheral blood mononuclear cells (PBMCs) from optic neuritis (ON) patients. Cells were divided into the normal control (NC), anti-CD3/CD28 stimulation (Anti-CD3/CD28), and anti-CD3/CD28 stimulation plus upadacitinib (UPA) groups (n = 6 per group).

(B-D) Proportions of CD3^+^CD8^-^IL-17A^+^ T cells (Th17), CD3^+^CD8^-^IFN-γ^+^ T cells (Th1), and CD3^+^CD8^-^CD25^+^FOXP3^+^ (Treg). PBMCs isolated from ON patients were cultured in vitro and assigned to three groups: NC, Anti-CD3/CD28 stimulation, and Anti-CD3/CD28 stimulation plus MCL1 inhibitor (MCL1i) (n = 6 per group).

(E-I) Flow cytometric detection of glycolysis-related molecules (HK2, PFKFB3, LDHA, GLUT1, and PKM2) in CD3^+^CD8^-^ T cells derived from ON patient PBMCs. In vitro cultured cells were categorized into NC, Anti-CD3/CD28 stimulation, and UPA treatment groups. Representative flow cytometry plots illustrate the percentages of CD3^+^CD8^-^HK2^+^, CD3^+^CD8^-^PFKFB3^+^, CD3^+^CD8^-^LDHA^+^, CD3^+^CD8^-^GLUT1^+^, and CD3^+^CD8^-^PKM2^+^ T cells in each group (n = 6 per group).
